# Protein Complex Interactor Analysis and Differential Activity of KDM3 Subfamily Members Towards H3K9 Methylation

**DOI:** 10.1371/journal.pone.0060549

**Published:** 2013-04-11

**Authors:** Michael Brauchle, Zhiping Yao, Rishi Arora, Sachin Thigale, Ieuan Clay, Bruno Inverardi, Joy Fletcher, Paul Taslimi, Michael G. Acker, Bertran Gerrits, Johannes Voshol, Andreas Bauer, Dirk Schübeler, Tewis Bouwmeester, Heinz Ruffner

**Affiliations:** 1 Developmental & Molecular Pathways, Novartis Institutes for Biomedical Research, Basel, Switzerland; 2 Center for Proteomic Chemistry, Novartis Institutes for Biomedical Research, Cambridge, Massachusetts, United States of America; 3 Friedrich Miescher Institute for Biomedical Research, Basel, Switzerland; Pohang University of Science and Technology (POSTECH), Republic of Korea

## Abstract

Histone modifications play an important role in chromatin organization and gene regulation, and their interpretation is referred to as epigenetic control. The methylation levels of several lysine residues in histone tails are tightly controlled, and JmjC domain-containing proteins are one class of broadly expressed enzymes catalyzing methyl group removal. However, several JmjC proteins remain uncharacterized, gaps persist in understanding substrate recognition, and the integration of JmjC proteins into signaling pathways is just emerging. The KDM3 subfamily is an evolutionarily conserved group of histone demethylase proteins, thought to share lysine substrate specificity. Here we use a systematic approach to compare KDM3 subfamily members. We show that full-length KDM3A and KDM3B are H3K9me1/2 histone demethylases whereas we fail to observe histone demethylase activity for JMJD1C using immunocytochemical and biochemical approaches. Structure-function analyses revealed the importance of a single amino acid in KDM3A implicated in the catalytic activity towards H3K9me1/2 that is not conserved in JMJD1C. Moreover, we use quantitative proteomic analyses to identify subsets of the interactomes of the 3 proteins. Specific interactor candidates were identified for each of the three KDM3 subfamily members. Importantly, we find that SCAI, a known transcriptional repressor, interacts specifically with KDM3B. Taken together, we identify substantial differences in the biology of KDM3 histone demethylases, namely enzymatic activity and protein-protein interactions. Such comparative approaches pave the way to a better understanding of histone demethylase specificity and protein function at a systems level and are instrumental in identifying the more subtle differences between closely related proteins.

## Introduction

Histones are the main building block of nucleosomes that structure DNA in the nucleus and regulate local accessibility to DNA [1]. The histones, and especially their N-termini, are highly modified by several different post-translational modifications, including acetylation, methylation, phosphorylation and ubiquitination, among others. These modifications not only play immediate roles in co-regulating gene transcription and chromatin organization but are also at the source of long-term epigenetic memory mechanisms [2]. This is because specific modifications are recognized by “reader” proteins that assemble relevant chromatin associated protein complexes that are responsible for the interpretation of histone modifications. Ultimately, the combination of these modifications represents an additional layer of information storage and this has been termed the “histone code” [3]. The resulting higher order chromatin composition can be inherited through cell division, remembering a cellular state, and this is reflected in the phenomenon of epigenetic inheritance [4]. However, there is a lot to be learned: only recently, a mass spectrometry-based approach identified additional types of modifications and increased the number of described histone modifications by about 70%, bringing their total number to well over 100 [5]. The biological significance of these recently identified modifications is not well understood, and it seems likely that there are still additional modifications to be discovered. In addition, many enzymes that add or remove these modifications not only remain to be identified but also their biological role, detailed mechanism of action, regulation, and influence on each other will have to be characterized in more detail to better understand epigenetic control.

Within euchromatin, the specific status of post-translationally modified histone tails orchestrates gene regulation by rendering a locus transcriptionally active or repressed [6]. For example, histone acetylation is generally observed in actively transcribed genes where it is neutralizing the positive charge of histones, thereby increasing the accessibility of DNA for additional factors. Other classes of histone modifications, for example lysine methylation, participate in activation and repression of gene expression depending on the specific residue on which they are encountered. Generally, nucleosomes decorated with methylated H3K4, H3K36 and H3K79 are indicative of active genes while methylation on H3K9, H3K27 and H4K20 are considered repressive marks. On a given lysine residue, it is the interplay between methyl transferases and demethylases that control the methylation level and thereby gene transcription and ultimately the cellular outcome. Histone lysine methylation is catalyzed by SET domain containing proteins and DOT1L homologues [7]. There are 2 classes of enzymes known that remove histone methylations through an oxidative mechanism [8]. LSD1 and LSD2 use FAD as cofactor and demethylate mono- and dimethylated lysines whereas Jumonji(Jmj)-C domain containing proteins use iron and α-ketoglutarate as cofactors and are also able to demethylate trimethyl- in addition to mono- and dimethyl-lysines [8]. There are 30 JmjC proteins in the human genome and 18 have been shown so far to possess Histone demethylase (HDM) activity [9].

Many cell types express a plethora of different JmjC domain containing proteins, and several of these proteins actually catalyze methyl group removal on the same lysine residues. However, the system is not overly redundant as individual demethylases are recruited to specific locations in the genome, affecting only a certain set of target genes. It is becoming clear that JmjC proteins are recruited to many genomic loci but their influence on specific gene expression levels is often relatively minor; indeed, they more likely act by fine-tuning gene expression [9]. HDM proteins can be further divided into subfamilies based on sequence homology. In general, members of the same subfamily demethylate the same lysine residue.

To address the functional specificity of different JmjC proteins, we decided to compare a whole subfamily of HDM's in the same cellular environment. To do so, we choose the KDM3 (KDM: Lysine demethylase, also known as JMJD1 or JHDM2) proteins KDM3A, KDM3B and JMJD1C. As compared to other HDM subfamilies, where many members are characterized, relatively little is known about the KDM3 members [9]. The KDM3 subfamily is evolutionarily conserved and has expanded, as compared to mice, to 6 members in *Arabidopsis thaliana* [10]. One of them is IBM1/JMJ25, and mutations in this gene result in increased methylation of H3K9methyl1 (me1) and -me2 and spreading of DNA methylation [11], [12]. While *C. elegans* lacks a KDM3 homologue, *Drosophila melanogaster* has a single KDM3 homologue, CG8165; its loss of function phenotype is not known but there is some evidence that it genetically interacts with Notch signaling [13]. Mammalian KDM3A is the best characterized KDM3 paralog, and it has been shown that KDM3A removes H3K9me1 and –me2 groups [14]. Knockout mice are viable but sterile and display an adult onset obesity phenotype [15], [16]. KDM3B has been suggested to be a candidate tumor suppressor gene [17]. JMJD1C has been described as an androgen receptor (AR)-interacting protein [18], and more recently, truncated mouse Jmjd1C has been proposed to be a H3K9me1/2 HDM [19]. In a fourth member of this subfamily, HAIRLESS, specific amino acids known to be important for enzymatic activity in other subfamily members have been replaced; since it is generally accepted that this abrogates HDM activity we are excluding this protein from our analysis.

Here we compare and contrast enzymatic activities and cellular interaction partner candidates of the three human KDM3 subfamily members in a common cellular environment. We show that wild-type KDM3A and KDM3B are H3K9me1/2 demethylases, report absence of enzymatic activity of JMJD1C and establish Suppressor of cancer cell invasion (SCAI) as a novel interaction partner of KDM3B.

## Results

### Enzymatic activity of KDM3 subfamily members: KDM3A and KDM3B are H3K9me1/2 demethylases while JMJD1C is not

**Figure 1:**
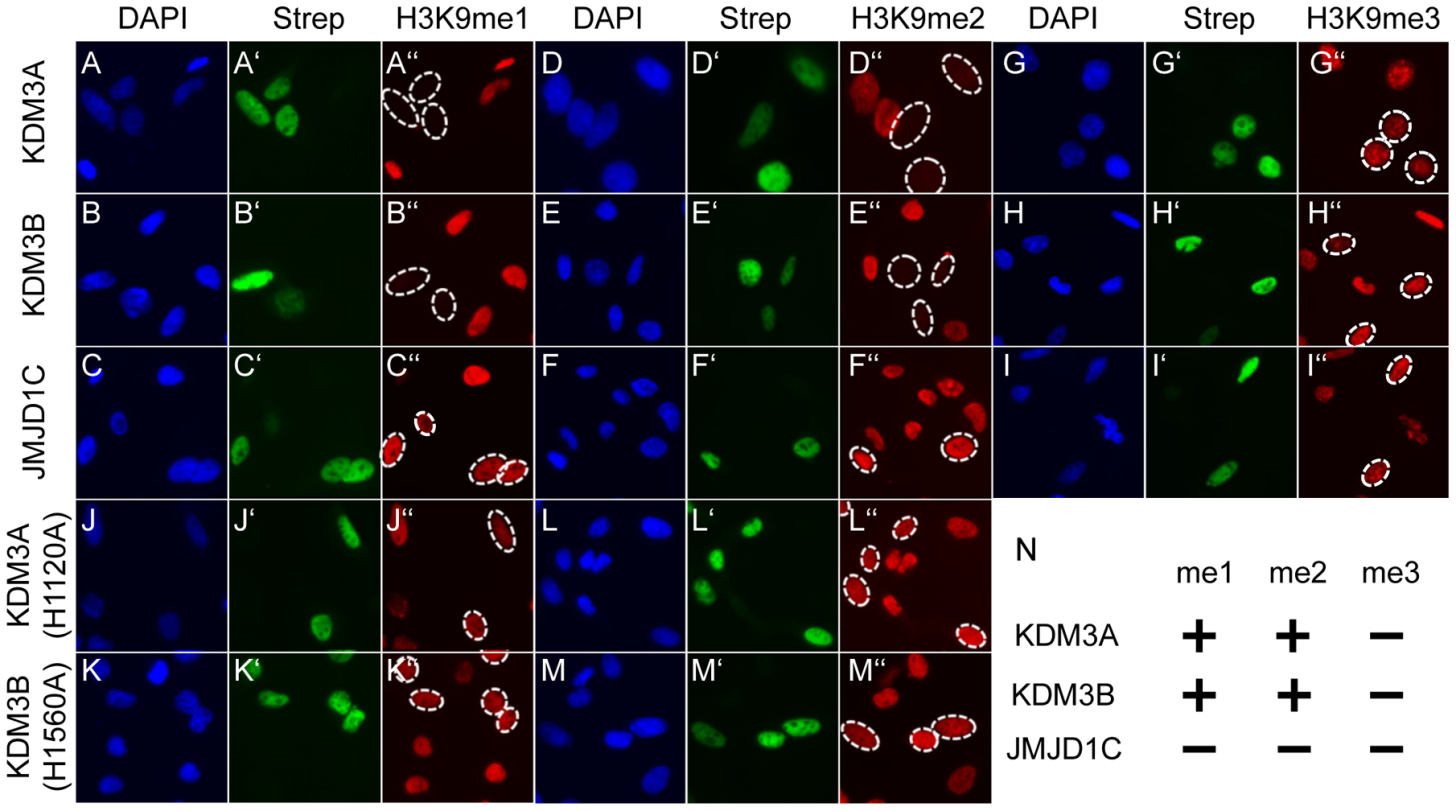
Enzymatic activity of KDM3 subfamily members towards H3K9 methylation. Individual KDM3 subfamily members were transiently overexpressed in U-2 OS cells. (A-M) DAPI staining indicating cell nuclei. (A'-M') Cellular expression of Avi-tagged KDM3 subfamily members, as detected by streptavidin-AlexaFluor-488 recognizing the biotinylated Avi-tag. (A’’-M’’) H3K9me1, -me2 or -me3 groups, respectively, as detected by antibody staining. White circles outline the transfected cells in the last panel of each series. Note that cells transfected with KDM3A and KDM3B (A, D, G and B, E, H) abolish H3K9me1 (A’’ and B’’) and -me2 (D’’ and E’’) but not -me3 (G’’ and H’’) staining. On the other hand, JMJD1C transfection (C, F, I) does not decrease H3K9me1 (C’’), -me2 (F’’) or -me3 (I’’) levels. The catalytic mutant versions of KDM3A(H1120A) (J, L) and KDM3B(H1560A) (K, M) neither reduce H3Kme1 (J’’, K’’) nor H3K9me2 (L’’, M’’) levels. N shows the summary of the enzymatic activity described above.

**Figure 2:**
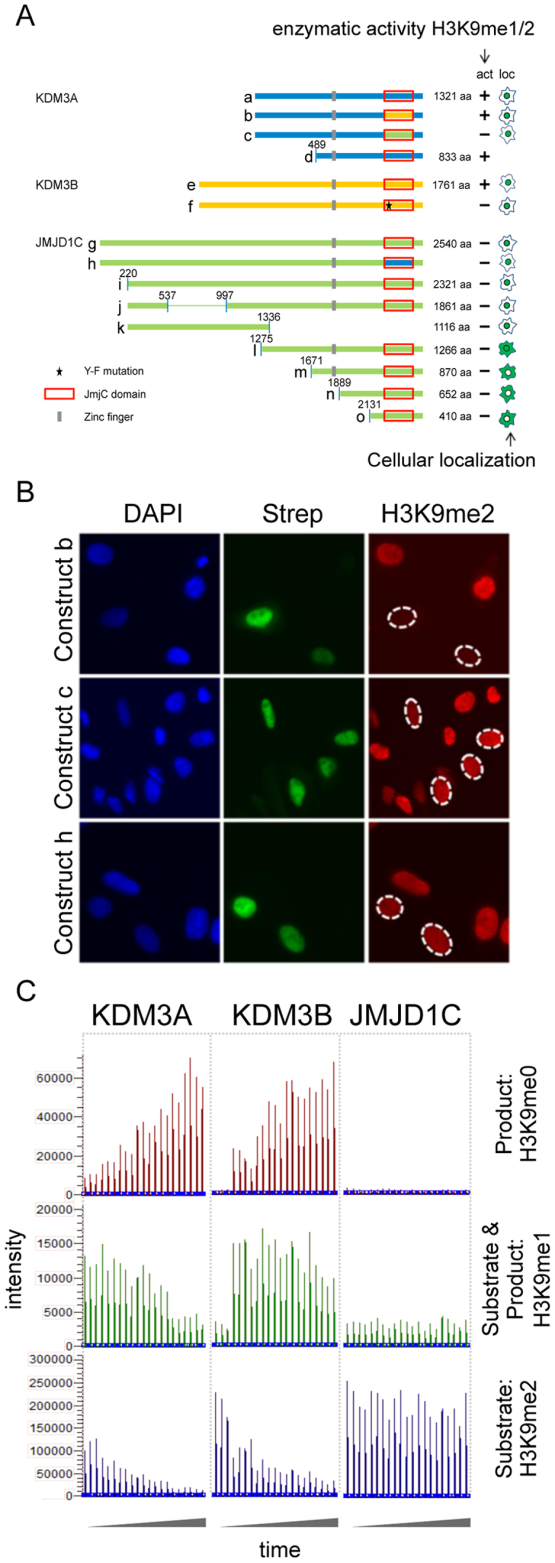
Domain mapping of KDM3 subfamily members identifies regions important for demethylase activity towards methylated H3K9. (A) Overview of constructs used in this study (left) and summary of results obtained for each construct with regard to demethylase activity towards H3K9 and subcellular localization (right). Full-length and truncated KDM3A (a and d, respectively) and full-length KDM3B (e) show activity towards H3K9me1 and -me2. Full-length and truncated versions of JMJD1C (g and i-o, respectively) do not show any enzymatic activity against either H3K9me1 or -me2. Construct i corresponds to the alternative splice isoform 2 of JMJD1C. Note that constructs d and m as well as e and j are similar in size, respectively. The star denotes the Y to F mutation in KDM3B (f), the red box denotes the JmjC domain in each construct, the grey box denotes the putative Zinc finger. (B) Hybrid constructs in which the JmjC domain in KDM3A was exchanged with the one of KDM3B (Construct b) or JMJD1C (Construct c) were assayed for their ability to demethylate H3K9me2 and –me1. Whereas construct b was active against both –me2 and –me1, construct c was inactive against both methyl groups. The hybrid construct in which the JmjC domain in JMJD1C was exchanged with the one of KDM3A (Construct h) can neither remove methyl group H3K9me2 nor –me1; only the data for –me2 are shown for either construct. (C) MS-based assessment of KMD3A, KDM3B and JMJD1C catalytic activity towards H3K9me2 and –me1. H3K9me2 peptides were incubated for 2 hours with the required co-factors and either recombinant KDM3A (aa511-1321), KDM3B(aa879-1761) or JMJD1C (aa1696–2540). Along H3K9me2 substrate, H3K9me1 and H3K9me0 reaction products were quantified using MS. Reactions were performed in triplicates, and H3K9me0, –me1 and –me2 levels were measured at 7 time intervals during the 2 hour incubation period, hence the 21 peaks shown per sample. Note that in the case of KDM3A and KDM3B, H3K9me2 levels strongly and H3K9me1 levels weakly drop during the incubation period, while H3K9me0 levels steadily increase over the course of the experiment. Using JMJD1C, neither H3K9me0 nor –me1 were produced over time up to the end of the 2 hour incubation period, indicating that JMJD1C cannot demethylate H3K9me1 or –me2.

We set out to identify the specificity of the three KDM3 subfamily members towards histone lysine residues. KDM3A was among the first JmjC domain-containing enzymes described with H3K9me1 and -me2 specificity [14]. Despite considerable differences in length, an amino acid alignment of the three KDM3 proteins shows that there are two regions with high similarity (Figure S1A). The first region encompasses a non-canonical C2HC4 zinc-finger domain which has been shown to be required for enzymatic activity of KDM3A [14]. The second region comprises the enzymatic 223–224 aa long JmjC domain which shows 64% overall aa similarity among KDM3 subfamily members. Pair-wise JmjC domain comparisons indicate that KDM3A and KDM3B harbor the most similar (86% aa similarity) JmjC domains. In addition, the catalytically important residues involved in co-factor binding during the oxidative demethylation reaction of JmjC proteins are fully conserved (Figure S1A) [20]. Therefore, we predicted that all three KDM3 proteins should be enzymatically active. All three are endogenously expressed in many cell lines, including human osteosarcoma U-2 OS cells [21]. To determine the effect of KDM3 subfamily members on methylation, we overexpressed individual proteins in this cell line to assay bulk changes in histone methylation levels. All three proteins were primarily localized in the nucleus with a broad nuclear distribution (Figure 1A', B' and C'). As expected, we confirmed that overexpression of KDM3A specifically reduced H3K9me1 and -me2 but not H3K9me3 levels, as assessed by methylation state-specific antibodies in immunocytochemistry analyses (Figure 1A”, D’’ and G’’). Similarly, we showed for the first time that full-length KDM3B demethylates H3K9me1/2 upon overexpression (Figure 1B’’, E’’ and H’’), as has previously been shown for a truncated version [22]. On the other hand, we did not observe H3K9 demethylase activity for JMJD1C (Figure 1C’’, F’’ and I’’). Next we tested additional methylation sites, including H3K4, K27 and K36 marks, as well as H4K18 and K20, but again JMJD1C overexpression did not result in visible changes of their methylation levels (Figure S2). To exclude a cell line specific effect, all overexpression analyses were also performed in the human embryonic kidney cell line HEK293T, where again KDM3A and KDM3B were enzymatically active while JMJD1C overexpression did not affect H3K9 methylation levels (Figure S3A–F). KDM3 subfamily members were further overexpressed in HeLa, NIH3T3, and TM3 cells, and again, the same results were obtained (Figure S3 G–N). In addition, we extended these observations to the second described splice isoform of JMJD1C which is 219 aa shorter than the first isoform (Figure 2A construct i; Figure S4 C–D). Finally, a full-length mouse Jmjd1C construct also failed to reduce H3K9 methylation levels upon overexpression (Figure S4 A–B). Taken together, these results show that overexpression of KDM3A and KDM3B strongly reduced global H3K9me1 and – me2 levels, while overexpression of JMD1C/Jmjd1c did not.

JmjC containing proteins function in an iron and α-ketoglutarate dependent mechanism [20]. It has been shown that single amino acid substitutions in the conserved active sites are sufficient to completely abrogate enzymatic activity, as shown for example for KDM7 [23]. To this end, we mutated one of the histidines involved in iron binding in the active site of KDM3A and B (KDM3A(H1120A) and KDM3B(H1560A)) to alanine, each, and tested the activities of these mutants towards H3K9me1/2. As expected, both proteins localized to the nucleus (Figure 1J', K', L' and M'). Indeed, overexpression of these mutants did not cause demethylation of H3K9 (Figure 1J’’, K’’, L’’ and M’’), suggesting that enzymatic activity occurs by the expected co-factor-dependent mechanism.

It has previously been suggested that a short version of mouse Jmjd1c is an active H3K9me1/2 demethylase enzyme [19]. Therefore, we performed several experiments to address this discrepancy compared to our observations described above. In our experiments, we used a full-length JMJD1C expression construct, and we noticed that overexpression of this construct resulted in lower protein levels compared to KDM3A and KDM3B, as judged by Western blot and ICC analyses, likely due to less efficient transfection and expression of the large JMJD1C isoform (Figure S5A). To generate JMJD1C species that express similar levels as KMD3A and KDM3B, we first generated a set of JMJD1C deletion constructs (Figure 2A, constructs i-o), including truncations that resulted in C-terminal JMJD1C fragments corresponding in size to KMD3A and KDM3B (Figure 2A, j and m). Since it had previously been shown that even a truncated version of KDM3A retains enzymatic activity [14], we also engineered a smaller KDM3A fragment (Figure 2A, d). Deletion of the N-terminal regions of JMJD1C resulted in loss of nuclear localization (Figure 2A, l-o; and Figure S6). To re-direct the localization of these truncated species, a heterologous nuclear localization signal (NLS), with or without a GFP fusion, was engineered to the N-termini of the JMJD1C fragments, thereby restoring nuclear localization (Figure S6). This set of constructs allowed us to compare side by side full-length and truncated KDM3A with similarly sized truncated JMJD1C to assess enzymatic activity towards H3K9me1/2. Western blot analyses revealed that the JMJD1C truncations expressed at similar levels compared to full-length KDM3A and KDM3B (Figure S5B). In agreement with our results depicted above and previous studies [14], full-length and truncated KDM3A efficiently removed H3K9me1/2. However, none of the JMJD1C species tested revealed any demethylation activity towards H3K9me1/2/3 (data summary presented in Fig. 2A; and Figure S4 E–Q).

**Figure 3:**
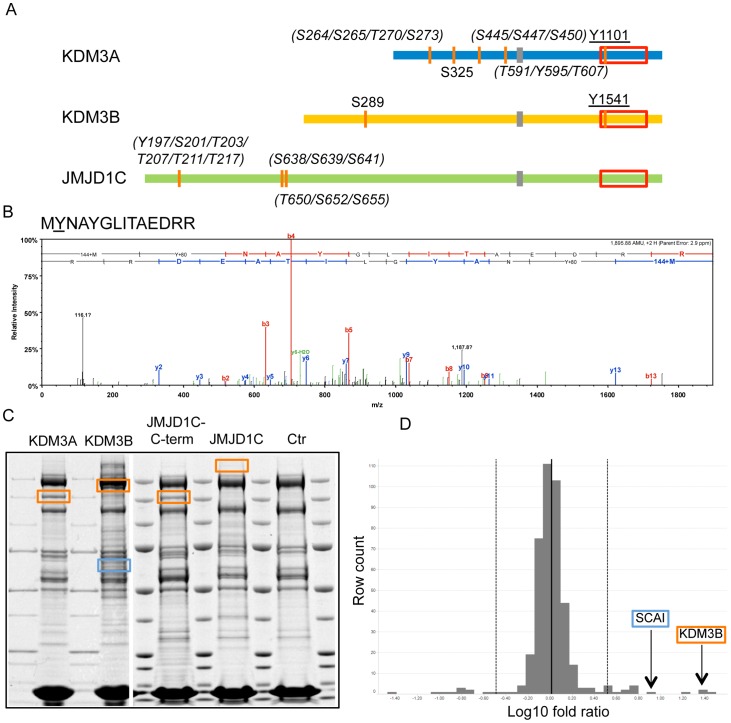
MS analysis of KDM3 subfamily members. (A) Phosphorylated peptides and residues identified in overexpressed KDM3A, KDM3B and JMJD1C using MS. Amino acid positions of the phosphorylated sites are indicated in the respective protein. Underlined phosphorylated sites are conserved. Potential phospho-sites within identified phospho-peptides are indicated in italics and brackets. (B) MS/MS spectra of the KDM3B peptide containing phosphorylated Y1541 (underlined). (C) Coomassie-stained gels showing affinity purifications of Avi-tagged, overexpressed KDM3 subfamily members from lysates of transfected HEK293T cells. The different lanes show individual purifications of KDM3A, KDM3B, JMJD1C C-term and JMJD1C as well as a control purification from mock-transfected HEK293T cells. The positions of the individually overexpressed proteins are indicated by orange squares, the position of the KDM3B interactor SCAI is indicated by a blue square. These samples were subjected to quantitative MS analysis. (D) Relative enrichment of KDM3B interactor candidates in relation to the mock control. The 406 proteins identified with at least 4 peptides were binned into 45 columns; stippled lines indicate 2 standard deviations from the mean. Proteins that centered around 0 were not enriched, whereas proteins retrieved on KDM3B that were enriched with ≥2 standard deviations (right stippled line) were considered KDM3B candidate interactors. KDM3B and its interactor candidate SCAI are indicated by arrows and boxed in the same color as in C.

Second, there was a recent report indicating that another JmjC-containing enzyme, PHF2, is only active upon phosphorylation by PKA [24]. Forskolin treatment, a chemical that activates PKA through increased cAMP levels, of JMJD1C overexpressing cells, however, did not alter H3K9me levels (Figure S7A); nor did treatment with PMA a chemical that activates PKC (Figure S7B). We therefore set out to identify phosphorylation events on KDM3A and KDM3B that could be important for enzymatic activity. Indeed, many phosphorylation sites have been reported on KDM3 family members [25]. To identify phosphorylated sites on KDM proteins in our system, we used affinity purification-mass spectrometric (AP-MS) analyses on overexpressed KDM3 subfamily members. We identified five phosphorylated peptides on KDM3A, two on KDM3B and three on JMD1C. For some of the peptides, we could identify the identity of the phosphorylated amino acid (Figure 3A and Figure S8). One of the phospho-sites in KDM3B, phospho-Y1541 (Figure 3B), and one phospho-peptide in JMJD1C (phospho-peptide amino acid 196–218) have not been reported before. Phospho-Y1101 in KDM3A and phospho-Y1541 in KDM3B are in a conserved position and located within the JmjC domain towards its N-terminal end, just a few amino acids upstream of the residues that constitute the enzymatically active domain. For the KDM4 subfamily of proteins, this region is known to be important for H3K9 substrate recognition [26]. Interestingly, this tyrosine residue is not present in JMJD1C (Figure S1A). To test whether a tyrosine at this site in KDM3B is important for enzymatic activity and/or substrate recognition, the activity of a KDM3B Y1541F mutation was tested upon overexpression. The mutant KDM3B was functional and could demethylate H3K9me1 and –me2 in our cellular system (Figure S9C–D). While these findings suggest that the presence and phosphorylation of KDM3B Y1541 is not necessary for the demethylation reaction *per se*, it could still be important for KDM3B targeting or be involved in signaling. We did not identify additional phosphorylation sites which are conserved in KDM3A and KDM3B but not in JMJD1C and which could explain the loss of enzymatic activity of the latter.

Third, we generated hybrid constructs in which we exchanged the JmjC domains among the three KDM3 proteins (Figure 2A, constructs b, c and h). All chimeric proteins remained localized to the nucleus. When the JmjC domain of KDM3A was exchanged by the JmjC domain of KDM3B, enzymatic activity towards H3K9me1 and –me2 was retained (Fig. 2B, construct b). On the other hand, when the JmjC domain of JMJD1C was introduced into the KDM3A backbone, enzymatic activity towards H3K9methylation was lost (Fig. 2B, construct c). Exchanging the JmjC domain in JMJD1C with the enzymatically active JmjC domain of KDM3A did not restore HDM activity (Fig. 2B, construct h). These data suggest that either the N-terminus of JMJD1C might negatively interfere with enzymatic activity of C-terminally fused active JmjC domains or that the N-termini of KDM3A and KDM3B but not JMJD1C contain domains important for enzymatic activity. In summary, both the sequence identity of the JmjC domain as well as the protein sequence N-terminal to the JmjC domain determine activity of the HDM proteins.

Fourth, as an alternative and complementary approach to overexpression in cellular systems, we set out to test HDM activity in a biochemical assay format. Multiple forms of human JMJD1C recombinant proteins were expressed in different systems, including full-length JMJD1C(1–2540) in insect and mammalian cells, truncated JMJD1C(1696–2540) in insect cells, and the JmjC domain of JMJD1C(2131–2540) in *E. coli*. Most of them were monomeric, as judged by size exclusion chromatography, but all failed to show demethylase activity against H3K9me1/2/3, using histone H3(1–21)K9me1/2/3 peptide substrates, despite significant attempts at reaction buffer optimization (Fig. 2C and data not shown). Meanwhile, KDM3A recombinant proteins were expressed in the same manner, including full-length KDM3A(1–1321) and truncated KDM3A(511–1321), which corresponds to JMJD1C(1696–2540). All of these KDM3A proteins show activity towards H3K9me1/2 performed side by side with JMJD1C proteins in the same biochemical assay (Figure 2C). In addition, also KDM3B(aa879–1761) showed enzymatic activity in our biochemical assay (Figure 2C). We also compared the phosphorylation status of KDM3A(511–1321) and JMDJ1C(1696–2540) recombinant proteins after purification from insect cells. We found no evidence of phosphorylation on KDM3A, while JMJD1C was highly phosphorylated. To exclude that phosphorylation would render JMJD1C inactive, we dephosphorylated JMJD1C(1696–2540) *in vitro* and tested its demethylase activity, but still the protein was inactive (Figure S10A).

Taken together, we report here that KDM3A and KDM3B are active H3K9me1/2 histone demethylases, whereas we found no evidence for enzymatic activity of JMJD1C towards H3K9me1/2/3.

### A single amino acid in KDM3A, T667, affects HDM activity towards H3K9me1 and –me2

JmjC domain proteins generally demethylate two of the possible three methylation states on a particular lysine residue. However, it is not well understood how substrate recognition and specificity between the different methylation states is achieved. In several cases, though, it has been shown that the JmjC domain alone is not sufficient to catalyze the demethylation reaction [27]. Therefore, we wanted to explore whether additional amino acid residues are important for enzymatic activity of the KDM3 subfamily and see if a lack of such residues in JMJD1C could possibly help to explain the absence of its enzymatic activity.

**Figure 4:**
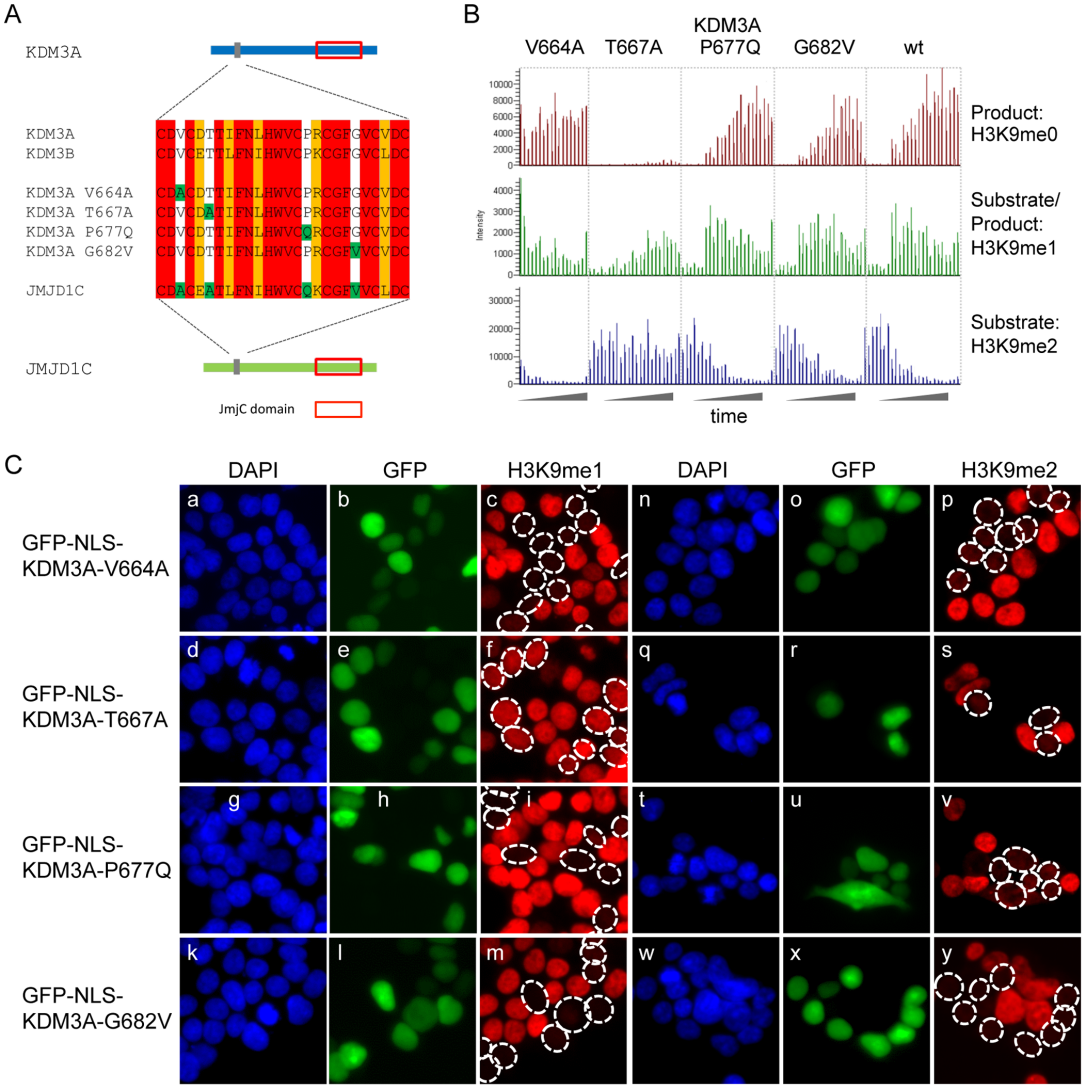
The Zinc finger mutant KDM3A T667A loses its ability to efficiently demethylate H3K9me1. (A) Sequence alignment of the three zinc finger domains of the KDM3 subfamily members. Amino acids marked in red are fully conserved between all three proteins, amino acids marked in orange are conserved in KDM3B and JMJD1C but not in KDM3A, and amino acids marked in white are conserved between KDM3A and KMD3B but not JMJD1C. The latter served as template to convert each amino acid in KDM3A to the corresponding amino acid present in the JMJD1C zinc finger domain, as indicated in green. (B) The four zinc finger mutants generated in KMD3A were analyzed for their ability to demethylate H3K9me1 and –me2 using a biochemical approach combined with a MS-based readout, similarly as described in Fig. 2C. KDM3A T667A revealed reduced activity towards H3K9me2 and strongly reduced activity towards H3K9me1 under the conditions tested. The other three zinc finger mutants behaved like wild-type KDM3A. (C) The same four zinc finger mutants were analyzed upon transient overexpression as GFP-NLS-fusion proteins in HEK293T cells for their ability to demethylate H3K9me1 and –me2. The following constructs were employed: a-c, n-p: GFP-NLS-KDM3A-V664A; d-f, q-s: GFP-NLS-KDM3A-T667A; g-i, t-v: GFP-NLS-KDM3A-P677Q; k-m, w-y: GFP-NLS-KDM3A-G682V. Lanes a,d,g,k and n,q,t,w: DAPI; lanes b,e,h,l and o,r,u,x: GFP to monitor transfected cells; lanes c,f,i,m and p,s,v,y: methylation state of H3K9me1 and -me2, respectively. GFP-NLS-KDM3A-T667A lacks activity against H3K9me1 but retains activity against H3K9me2 (f and s), while the other three mutants are active against both H3K9me1 and –me2 (c,i,m and p,v,y).

The JmjC domain swap experiments (Figure 2) suggested two features; first, that the JmjC domain of JMJD1C is non-functional if placed into the heterologous KDM3A context, and second, that the JMJD1C N-terminal part inhibits the otherwise active JmjC domain of KDM3A in the JMJD1C backbone. To follow-up on this observation, we turned our attention to the only other known domain of KDM3A important for enzymatic activity, the non-canonical C2HC4 zinc finger domain [14]. An alignment of this domain of KDM3A, KDM3B and JMJD1C identified 4 amino acids which are identical in KDM3A and KDM3B but different in JMJD1C (Figure 4A). First, we exchanged the C2HC4 zinc finger domain of JMJD1C with the corresponding domain of KDM3A. However, despite the change in the zinc-finger JMJD1C remained inactive in the biochemical assays (Figure S10B). Since it has been shown that this domain is necessary for enzymatic activity in KDM3A we next individually mutated the four amino acids in KDM3A to be identical to the corresponding amino acids in JMJD1C to assess whether one of these amino acids plays a role in enzymatic activity. We then tested the activity of these KDM3A V664A, T667A, P677Q and G682V mutants towards H3K9 methylation in biochemical (Figure 4B) and cellular assays upon overexpression (Figure 4C). Interestingly, one of these mutants, T667A, remains active against H3K9me2 but poorly demethylates H3K9me1, if at all, as evident in both cellular and biochemical assays (Figure 4B, C). Therefore, the threonine residue 667 in wild-type KDM3A is important for the execution of the catalytic demethylase activity towards mono H3K9 substrates. The other three mutants, V664A, P677Q and G682V, retain enzymatic activity against both H3K9me1 and –me2 (Figure 4B, C), indicating that these three amino acid residues do not contribute to enzyme specificity at H3K9me1 and –me2. In agreement with substituting the whole zinc-finger, reversibly substituting the corresponding amino acid of KDM3AT667 in JMJD1C, A1851, with a threonine residue does not restore enzymatic activity of JMJD1C (Figure S10C), suggesting that mutating this amino acid is not sufficient to explain the lack of enzymatic activity of JMJD1C. Furthermore, T1851 in a hybrid JMJD1C construct in which its JmjC domain has been replaced by the one of KMD3A does not show enzymatic activity, either (Figure S9A–B). Taken together, we show that in KDM3A T667 is important to differentiate H3K9me1 and -me2 but that mutating the corresponding aa in JMJD1C does not rescue its absence of enzymatic activity.

### The incorporation of KDM3 family members in the cellular environment

Multi-protein complexes are involved in the precise modulation of gene expression, and several HDM's have been shown to be integral members of such complexes in certain cell types. Apart from interactions with nuclear hormone receptors [14], [18], it is not known in which context KDM3 subfamily members function. Moreover, it is believed that the loss of one HDM family member might be compensated by the other family members [28]. If this were to be true, one might expect a good overlap of protein-protein interaction partners and/or a transcriptional dependency. To start to address the question of whether different KDM3 members recruit individual protein interaction partners to achieve transcriptional specificity, we wanted to know if they influence each other's transcription and what their protein-protein interaction partners are.

First, we used qRT-PCR analysis to determine knock-down efficiency of KDM3 subfamily members upon siRNA treatment in HEK293T cells. After 72 hrs of siRNA treatment mRNA levels were 19%, 12% and 28% of control levels for *KDM3A*, *KDM3B* and *JMJD1C*, respectively. Despite significant efforts, we did not identify si- or shRNA reagents that reduced *JMJD1C* mRNA levels below 25% of control levels (data not shown). We then tested by qRT-PCR if knockdown of individual subfamily members affected the expression of the other subfamily members. We found this not to be the case, suggesting that the three genes do not influence each others expression (Figure S11).

Next, we wanted to test if KDM3A and KDM3B reveal interaction partner specificity and offset that against the enzymatically inactive JMJD1C. To this end, we made use of a quantitative MS-AP approach. cDNAs encoding individual Avi-tagged members of the KDM3 subfamily were transiently co-expressed with IRES-driven bacterial biotin ligase (IRES-BirA), each, in HEK293T cells. As controls, the same amount of empty plasmid containing IRES-BirA was transfected in parallel into HEK293T cells. 72 hours following transfection, cell lysates were prepared, and protein complexes were immunoprecipitated using streptavidin-coupled beads. Following SDS gel electrophoresis, proteins were visualized using coomassie staining (Fig. 3C). We then employed state-of-the-art quantitative MS, where tryptic peptides of the different purifications were first labeled with the respective iTRAQ reagents [29]. Labeled tryptic peptides isolated from corresponding gel bands of the different KDM member purifications and control purifications were subsequently pooled and subjected to quantitative mass spectrometric analysis [29]. The abundance of iTRAQ labeled peptides identifies the relative protein abundance from each purification, providing a quantitative measure of the individual protein interaction partners. Due to the difficulty of overexpressing full-length JMJD1C, we also subjected an Avi-tagged JMJD1C truncation similar in length to KDM3A for interactor analysis. A nuclear localization signal (NLS) was fused to the latter construct to ensure nuclear localization (Figure S4 L–M). This NLS-JMJD1C-C-term protein co-precipitated three KPNA proteins among the top 6 identified interactors (Table S1). KPNA proteins interact with the NLS sequence [30] and thereby served as positive controls for our approach.

**Figure 5:**
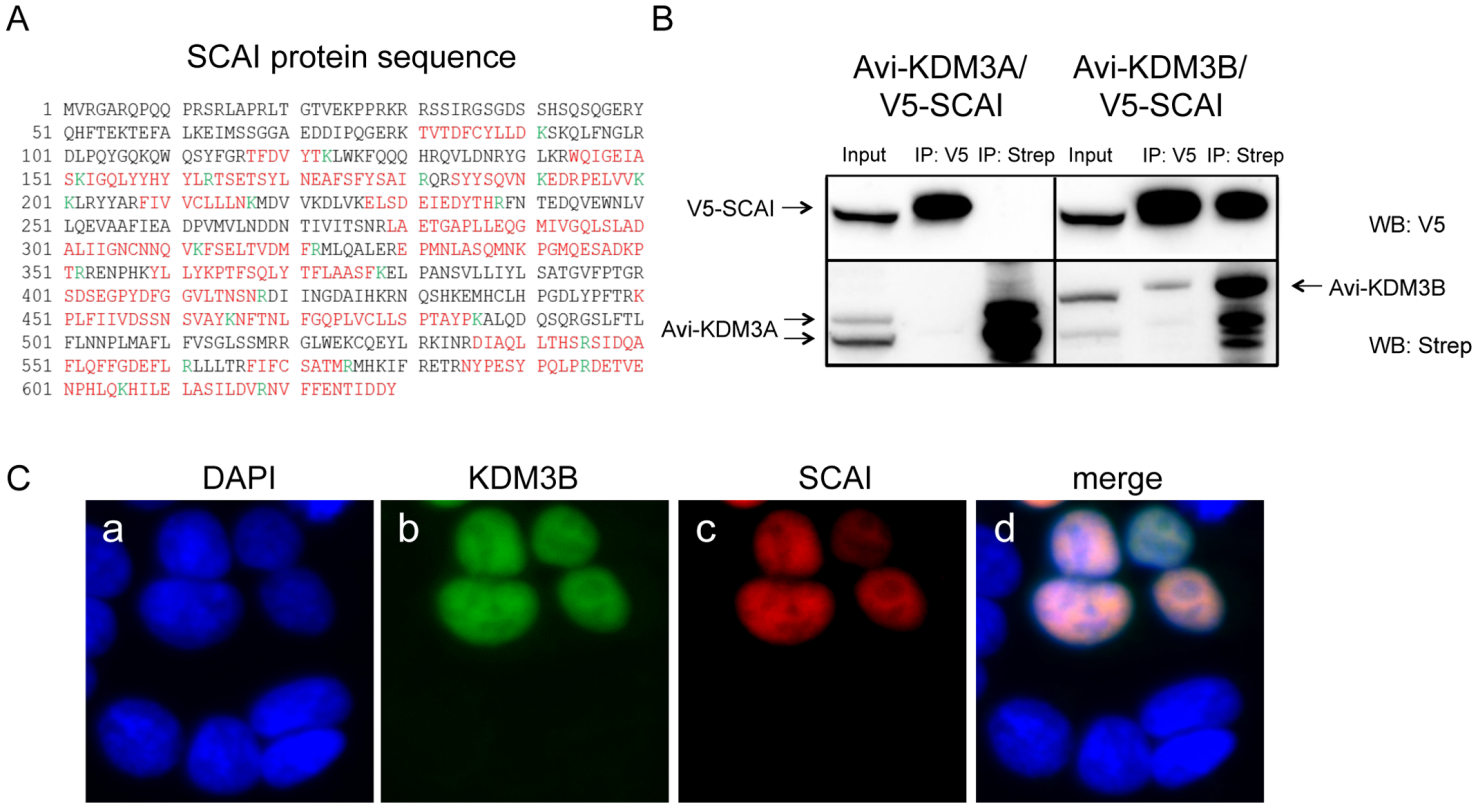
SCAI is a specific interactor candidate of KDM3B. (A) SCAI protein sequence with the peptides identified by MS highlighted in red. The amino acids marked in green indicate trypsin cleavage sites. SCAI sequence coverage by MS was 51%. (B) Reciprocal co-immunoprecipitation of SCAI and KDM3B. V5-SCAI was either co-expressed with Avi-KDM3A or Avi-KDM3B. Reciprocal co-immunoprecipitations using V5- antibodies or streptavidin-coated beads were performed and the immunoprecipitated proteins from each immunoprecipitation were separated on SDS gels. A V5-antibody and streptavidin-HRP were used to detect SCAI and KDM3A or KDM3B, respectively. Only KDM3B but not KDM3A co-precipitated with and was able to precipitate V5-SCAI, respectively. (C) Sub-cellular co-localization of KDM3B and SCAI in HEK293T cells. Avi-KDM3B and V5-SCAI were co-expressed in HEK293T cells and detected by immunoreagents against their respective tags (b and c). The two proteins were found to co-localize in the nucleus (d).

As expected, KDM3A, KDM3B and JMJD1C were among the most enriched proteins in each purification, respectively. For this analysis, we defined interactor candidates as proteins enriched on KDM3A or KDM3B by at least one standard deviation compared to the negative control, each, in two independent quantitative AP-MS experiments, respectively. Comparing the resulting interactomes with each other, we identified only a couple of common interaction candidates among KDM3 subfamily members (Table S1). Interestingly, we retrieved several interaction partner candidates specific for a particular KDM3 subfamily member. For example, the procollagen-lysine dioxygenases PLOD1, PLOD2 and PLOD3 were specifically retrieved on KDM3B. On the other hand, the suppressor of G2 allele of SKP1 homolog (SUGT1) was specifically retrieved on KDM3A. Most notably, SCAI was another protein which co-purified with KDM3B. SCAI was identified by multiple peptides covering more than 50% of the whole protein sequence (Figure 5A). We chose SCAI for follow-up interactor validation because it had previously been shown to be a transcriptional repressor involved in the suppression of cancer cell invasion, hence its name SCAI [31]. To verify SCAI as an interaction partner of KMD3B, we performed reciprocal co-immunoprecipitation experiments using V5-tagged SCAI and Avi-tagged KMD3A and KDM3B proteins. Confirming the data of the AP-MS analysis, SCAI was only pulled down with KDM3B but not KDM3A (Figure 5B, top). Importantly, SCAI was able to co-immunoprecipitate KDM3B but not KDM3A, validating SCAI as a specific interaction partner for KMD3B (Figure 5B, bottom). Moreover, exogenously expressed, tagged KDM3B and SCAI both co-localized in the nucleus (Figure 5C). These results indicate that KDM3 subfamily members have specific interaction partners, possibly explaining some aspects of their individual functions.

## Discussion

### No evidence for JMJD1C histone demethylase activity towards H3K9

Both cell-based and biochemical approaches failed to detect enzymatic activity of JMJD1C (Figures 1 and 2). The amino acid sequence of its JmjC domain includes the conserved residues known to be important for enzymatic activity and suggests it to be an active demethylase. A truncated mouse Jmjd1C version of the protein had been reported to be an active H3K9me1/2 HDM [19], however, in our hands the same construct was not active and possibly a different experimental set-up can explain this discrepancy. Our results suggest that JMJD1C is not an active H3K9 HDM, unlike its two other subfamily members.

While our data suggest that JMJD1C does not act directly as a H3K9 HDM, it nevertheless might be involved in regulating transcription and/or other cellular processes. Firstly, JMJD1C could, unexpectedly, act on a different lysine residue than H3K9. While we tested if JMJD1C demethylates other commonly methylated histone lysine residues, including H3K4, H2K27 and H3K36, there remain additional residues that are poorly characterized or where methyl-specific antibodies are not currently available. Secondly, JMJD1C might require an additional co-factor(s) that, if not co-expressed, cannot generate HDM activity, as judged by global assessment of H3K9 demethylation. For example, PHF2 has been reported to lack enzymatic activity upon overexpression unless PKA is artificially activated and in turn phosphorylates PHF2 [24]. However, we do not currently know if such an additional protein is needed; candidate interactors identified in our MS approach could prove useful to address this question. Thirdly, it is possible that JMJD1C acts exclusively on non-histone proteins. There are several JmjC proteins known which remove methyl groups on proteins other than histones. For example, FBXL11 has been shown to demethylate p65, thereby regulating the NF- κ B pathway [32]. In addition, JMJD6 has been shown to hydroxylate the splicing factor U2AF65 [33] while its role in histone demethylation is controversial [34], [35]. Fourthly, JMJD1C's predominant role could encompass a scaffolding function, its large size allowing a number of potential binding partners. Similar observations have been made for other JmjC proteins, e.g. for HAIRLESS the fourth member of the KDM3 subfamily or for JARID2, a protein involved in gene regulation through interaction with PRC2, both lacking enzymatic HDM function due to the loss of critical residues for co-factor binding within their JmjC domain [36], [37]. In addition, JMJD3 has been shown to play a role in chromatin remodeling independent of its H3K27 HDM activity [38]. Also, other epigenetic enzymes function in a similar manner, e.g. a mutant version of DNMT1 plays a role in gene transcription even though it is catalytically dead, hinting at scaffolding functions apart from methyltransferase activity [39]. In addition, DNMT3L is important for the regulation of DNA methylation through interactions with other DNMT3 proteins but has itself no DNA methyltransferase activity [40]. Interestingly *Arabidopsis thaliana* has 6 members of the KDM3 subfamily where two have lost conserved iron- and α-KG-binding amino acids [10], suggesting additional roles for KDM3 subfamily members independent of direct demethylation activity. Future studies will have to identify potential non-histone targets of JMJD1C and/or establish its role as scaffolding protein.

### KDM3T667 directs H3K9me1 and –me2 HDM activity

Structural studies have started to unravel the catalytic mechanism and the substrate specificity of certain JmjC proteins [27]. Explanations have been put forward why none of the JmjC proteins described so far can demethylate all three methylation states on the same lysine residue. For example, it has been suggested that PHF8 cannot demethylate trimethylated H3K9 due to steric hindrance, as the trimethylated peptide cannot fit into the active site [41]. On the other hand, it is believed that monomethylated H3K9 is not demethylated by KDM4A because the single methyl group cannot reach sufficient proximity to the iron ion, likely due to water molecules that direct the methyl group away from the hydroxylation site [42]. It is less understood how differentiation between the two other methyl substrates is achieved. Here we have identified an amino acid, T667, which contributes to the H3K9me1/2 substrate specificity of wild-type KDM3A (Figure 4). Threonine residue 667 could in theory act as a phospho-acceptor to modulate substrate specificity, however we have not found any evidence of T667 phosphorylation. Mutation of T667 to A667 alters specificity towards H3K9me2. Therefore, KDM3A T667 seems capable of aligning the methyl group of monomethylated H3K9 correctly in the active center, presumably bringing it in close proximity to the iron so that the reaction can be catalyzed. To our knowledge, this is the first time that a HDM mutation has been identified that preferentially affects the demethylation efficiency of one of its two natural substrate methyl groups under the experimental conditions applied. However, there are wild-type JmjC proteins which naturally only demethylate a doubly methylated lysine residue, for example PHF2 [24] or JMJD5 [43], restricting their HDM activities to only one of the three methylation states on a particular lysine residue. Moreover, the fact that T667 of KDM3A is not conserved at the corresponding position in JMJD1C could be one reason why JMJD1C is unable to demethylate H3K9me1. It should be noted that the putative zinc finger region is conserved among JMJD1C homologs in other species. Taken together, our findings could pave the way to develop specific low molecular weight inhibitors that prevent HDM activity towards a subset of methyl group substrates only. It will be interesting to elucidate the structure of the active domain of KDM3 proteins in order to get a better molecular understanding of the mechanism.

### Towards a description of the cellular role of KDM3 subfamily members

In general, chromatin modifying enzymes act in large protein complexes bound to chromatin to regulate transcriptional events. Individual protein complex members execute distinct functions as part of the whole chromatin modifying protein complexes. Until now, very few protein interaction partners of KDM3 subfamily proteins have been identified. JMJD1C was initially identified using yeast two-hybrid screens as a thyroid hormone receptor-interacting protein TRIP8 [44] and has later been shown to interact with the AR [18]. KDM3A has been shown to regulate AR target genes [14]. Here, we used a quantitative proteomics approach to identify specific interactor candidates of the KDM3 subfamily members (Figure 3). For comparative reasons, the experiments were carried out in the same cellular context. We have obtained very little overlap of putative interaction partners for each of the individual KDM3 subfamily members. We found only HSP8 and TRAP1 as putatively interacting with both KDM3A and KDM3B. While KMD3A and KDM3B proteins are enzymatically active in HEK293T cells, some interaction partners may not or only weakly be expressed in these cells, precluding their identification by mass spectrometric approaches. A lack of multiple common interaction partners would argue against highly redundant functions among these two KDM3 proteins, at least under the experimental conditions applied. It has previously been shown that other HDM subfamilies function in different cellular contexts. For example, KDM5 subfamily members are part of several different protein complexes; KDM5A interacts with the PRC2 complex [45], KDM5B with the NuRD complex [46], KDM5C forms a complex with REST and HDAC1 and HDAC2 [47], and KDM5D has been found to interact with RING6A, a polycomb-like protein [48]. In these cases, though, KDM5 subfamily members were purified from different cell types.

Another unresolved question is how the KDM3 subfamily members are recruited to chromatin. For example, we identified certain ARID proteins known to bind AT rich DNA sequences [49] as putative KDM3 interaction partners, and future experiments will be necessary to see if they are involved in KDM3 recruitment to chromatin.

Importantly, we have identified SCAI as a specific interactor of KDM3B (Figure 5). In independent reciprocal co-immunoprecipitation experiments, we confirmed that SCAI co-precipitates with KDM3B but not KDM3A and *vice versa*. SCAI is a highly conserved protein ranging from mammals to *D. melanogaster* and plants. In mammals SCAI acts as a transcriptional repressor in the RhoA-Dia1 signal transduction pathway, where it has been shown to regulate cell invasiveness through upregulation of β-integrin [31]. We hypothesize that SCAI acts as transcriptional co-regulator in the context of KMD3B. Future studies will demonstrate how protein complexes containing SCAI and KDM3B regulate target gene expression.

Here, we started to unravel the complex cellular functions and specific interaction partners of the KDM3 subfamily of HDM's. We showed that KDM3A and KDM3B harbor H3K9me1/2 HDM activities, while JMJD1C did not. Indeed, while we were finishing this study, a manuscript has been published describing a short version of KDM3B as a H3K9 me1/2 HDM [22], supporting the notion that subfamily members share substrate specificity [9]. Furthermore, we identified putative novel interaction partners for all KDM3 subfamily members. Taken together, the comparative approach described in this work has significantly contributed to the increased molecular understanding of enzyme substrate and interaction partner specificity of the KDM3 subfamily members. Similar studies using other HDM subfamily members will further help to get a better understanding of the molecular networks in which HDM's and other chromatin modifying enzymes and transcription factors act together to orchestrate regulation of gene expression. These insights will be crucial in order to develop targeted therapies against diseases that have underlying causes in genetic perturbations of these systems.

## Materials and Methods

### Cell culture

HEK293T cells (ATCC CRL-11268) were cultured in DMEM GlutaMAX (GIBCO) containing 10% FBS (GIBCO). U-2 OS cells were cultured in DMEM/F12 (GIBCO) containing 10% FBS.

### Constructs

Individual KDM3 constructs were cloned into a N- or C-terminal Avi-tag expression vector containing an IRES-BirA using the Gateway cloning system (Invitrogen). The sequences cloned correspond to the coding regions of NM_018433.5 for KDM3A (Fig. 2, construct a), NM_016604.3 for KDM3B (Fig. 2, construct e), NM_032776.1 (Fig. 2, construct g) and NM_004241.2 (Fig. 2, construct i) for JMJD1C. Deletion constructs were engineered with Phusion Hot Start High Fidelity DNA polymerase (Finnzymes). Point mutations were introduced using the QuikChangeII XL kit (Stratagene). GFP-NLS-KDM3 constructs were generated by Gateway-mediated cloning of corresponding KDM3 regions 3′ to a GFP-NLS sequence in an engineered pcDNA3 vector. SCAI was cloned using Multiscribe reverse Transcriptase (Applied Biosystems) from HEK293T purified mRNA using the following primers: F: GGGGACAAGTTTGTACAAAAAAGCAGGCTTCatggtcagaggagcccgg and R: GGGGACCACTTTGTACAAGAAAGCTGGGTCttaatagtcatcaatggtattctcaaa. The resulting gene contains 1821bp, identical to NM_001144877.2, and was Gateway-cloned into the N-terminal Lumio-V5 vector (Invitrogen).

### Recombinant proteins

Full-length KDM3A and JMJD1C cDNAs in pENTR221 were Gateway-cloned into pDEST10 and pDEST26 (Invitrogen). Truncated KDM3A(aa511-1321), KDM3B(aa879-1761) and JMJD1C(aa1696-2540) were cloned into pFastBacHT_B vector (Invitrogen). Baculoviruses were generated using the Bac-to-Bac method from pDEST10 or pFastBac plasmids. For mammalian expression systems, HEK293-freestyle cells (Invitrogen) were used for transient expression of full-length JMJD1C proteins. Cell pellets containing recombinant proteins were lysed and cleared before loading onto affinity columns, purifications were achieved using His- or Flag-tag purifications followed by a desalting step prior to buffer exchange. The final buffer for protein was 25 mM Tris pH 7.5, 150 mM NaCl, 1 mM TCEP and 10% glycerol.

### Biochemical assays

Methylated H3K9me1, H3K9me2, H3K9me3 peptides were purchased from AnaSpec. The assay buffer contained 1 µM methylated peptide, 10–100 nM of the respective KDM3 enzyme, 20 mM HEPES pH pH 7.5, 1 mM α -ketoglutarate, 2 mM ascorbic acid, 40 µM FeSO4, 3 mM MgCl, 0.1% BSA and 0.01% Tween. Reactions were quenched with an equal volume of 20% acetic acid at different time-points between 0–120 minutes. LC-MS was used to follow both the depletion of substrate and generation of product.

### Immunofluorescence analyses

Sub-confluent cells were split 1:10 into poly-L-Lysine (Cultrex)-coated 96-well plates. On the next day, cells were transfected with 0.2 µg of the corresponding DNA using Lipofectamine 2000 (Invitrogen), according to the manufacturer's protocol. For Avi-tagged constructs, cells were treated with 225 nM biotin (Sigma). 24 hours later, cells were washed with PBS and fixed with 4% formaldehyde in PBS for 10 minutes. Cells were washed twice with PBS, then permeabilized and blocked for 1 hour with 0.2% triton X-100, 10% FBS in PBS. Cells were then incubated with the respective primary antibodies in 0.1% triton X-100, 5% FBS in PBS for 2 hours. Secondary Cy3-linked α-mouse and α-rabbit antibodies (GE Healthcare) were used at 1:750 dilutions during a 2 hour incubation. Streptavidin-coupled to AlexaFluor-488 (Molecular Probes, 1:1000) identified cells containing the Avi-tag expression constructs. After one PBS wash, cells were incubated for 10 minutes with DAPI (PromoKine) before they were washed again 2 times with PBS. The following primary antibodies were used: H3K9me1: Abcam ab9045; H3K9me2: Abcam ab1220; H3K9me3: Cell Signaling Technology 9754S. Images were taken on an Olympus microscope and processed using ImageJ (National Institutes of Health, imagej.nih.gov).

### Affinity purification and quantitative MS analysis (AP-MS)

Individual KDM3 subfamily members were overexpressed in HEK293T cells using an adapted version of the calcium phosphate method [50]. Briefly, cells were transfected at 40% confluency and incubated overnight at 3% CO_2_. In the morning of the following day, the transfection media was replaced with fresh media containing 225 nM biotin, and cells were incubated in 5% CO_2_ for another 48 hours. Cells were then washed twice with ice-cold PBS and scraped off before being snap-frozen in liquid nitrogen. Cells were incubated in lysis buffer (50mM Tris-Cl pH 7.4, 100 mM NaCl, 5% glycerol, 1.5 mM MgCl2, 1mM Na3VO4, 0.4% NP40, 25 mM NaF, 10 nM Calyculin A, 1 mM DTT, Protease inhibitors (complete protease inhibitor cocktail, Roche) and 0.2 mg/ml DNAseI (Sigma) for 30 minutes at 4°C. Lysates were first cleared by centrifugation and then incubated with high capacity streptavidin agarose (Thermo Scientific) for 2 hours. Beads were washed in lysis buffer without DNAse and eluted by boiling for 10 minutes in 2X LDS loading buffer (Invitrogen) supplemented with β-Mercaptoethanol. Appropriate amounts of eluates were then loaded onto 4–12% NuPage Gels (Invitrogen), and gels were stained with commassie brilliant blue G (Sigma). Lanes were cut into 16 consecutive pieces, proteins in each gel band trypsinized and labeled with the iTRAQ reagent. Corresponding samples from lanes of control and KDM3 purifications were then pooled. Tryptic peptides were separated by online nano-high pressure liquid chromatography (Eksigent, Dublin, CA) on a C_18_ reversed phase column (Magic 3-μm 100-Å C_18_ AQ; Michrom, Auburn, CA), using an acetonitrile/water system at a flow rate of 200 nl/min, prior to analysis on an LTQ Orbitrap Velos analyzer (Thermo Electron, Bremen, Germany). Tandem mass spectra were acquired in a data-dependent manner. Typically, 10 MS/MS measurements were performed after each high accuracy spectral acquisition range survey, and both HCD and CID tandem spectra were acquired. RAW MS files were converted to peak lists using Mascot Distiller (version 2.4.0.0), with spectrum merging enabled. The human portion (taxonomy ID: 9606) of the IPI data base version 3.87 (91′491 sequences of which 810 are common contaminants) was interrogated using the Mascot search algorithm [51]. One failed trypsin cleavage was allowed per search. The precursor and fragment ion tolerances were set to 10 ppm and 0.8 Da, respectively. Fixed modifications included the iTRAQ reagent (K, N-term) and Carbamidomethyl (C). Variable modifications included Oxidation (M), deamination (NQ) and pyroglutamic acid. After the database search, iTRAQ reporter ions were extracted, summed and normalised using an in-house algorithm. Only proteotypic peptides were used for protein quantitation.

### Co-Immunoprecipitation and Western Blot

HEK293T cells were cotransfected with Avi-tagged KDM3A or B and V5-tagged SCAI using the calcium phosphate method described above. Cells were treated and lysed as described for AP-MS experiments and split for incubation with either Streptavidin- or V5-agarose beads (Sigma). Co-immunoprecipitation reactions were eluted in 2X LDS loading buffer (Invitrogen) and subjected to standard SDS-PAGE and subsequent Western Blot analyses. Immunodetection reagents used were α-V5 (Invitrogen) in conjunction with α-mouse-HRP (GE Healthcare) to detect V5-SCAI, and Streptavidin-HRP (Pierce) to detect Avi-KDM3A or B. Protein bands were visualized using ECL+ (GE Healthcare).

## Supporting Information

Figure S1 **Amino acid alignment of KDM3 subfamily members.**
(TIF)

Figure S2 **Analysis of additional methyl marks upon overexpression of JMJD1C.**
(TIF)

Figure S3 **Enzymatic activity of full-length KDM3 subfamily members towards H3K9 methylation in HEK293T, HeLa, TM3 and NIH3T3 cell lines.**
(TIF)

Figure S4 **Enzymatic activity of mJmjd1c, as well as KDM3A and hJMJD1C deletion constructs towards H3K9 methylation.**
(TIF)

Figure S5 **Avi-KDM3A, -KDM3B and –JMJD1C levels, including certain deletion constructs, upon overexpression in HEK293T cells.**
(TIF)

Figure S6 **Sub-cellular localization of JMJD1C deletion constructs.**
(TIF)

Figure S7 **Lack of enzymatic activity of JMJD1C overexpression upon treatment with kinase activators forskolin and PMA.**
(TIF)

Figure S8 **Detection of phosphorylation events in KDM3 subfamily members.**
(TIF)

Figure S9 **Enzymatic activity of mutated KDM3 subfamily members towards methylated H3K9.**
(TIF)

Figure S10 **Lack of enzymatic activity of additional JMJD1C constructs in the biochemical assay.**
(TIF)

Figure S11 **No effect on KDM3 subfamily member gene expression upon reciprocal subfamily member gene knockdown.**
(TIF)

Table S1 **Protein interaction candidates of KDM3 subfamily members as identified using quantitative AP-MS.**
(XLSX)
