# Supplementary figures and images for: Protein Complex Interactor Analysis and Differential Activity of KDM3 Subfamily Members Towards H3K9 Methylation
Source: PLoS One. 2013 Apr 11;8(4):e60549. doi: 10.1371/journal.pone.0060549 (PMC3623819; doi:10.1371/journal.pone.0060549)

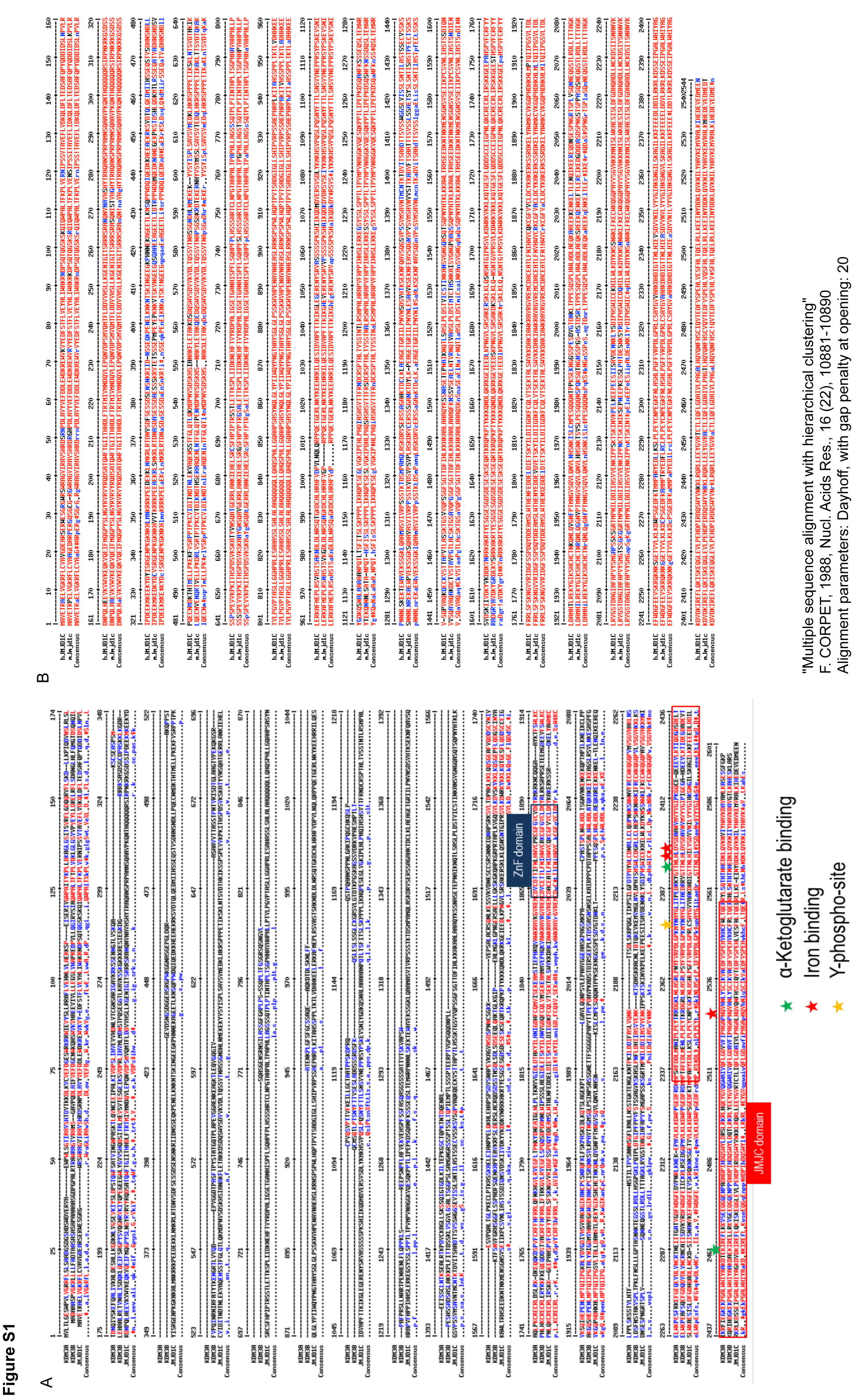

Supplement: Figure S1 — Amino acid alignment of KDM3 subfamily members. (TIF) [file pone.0060549.s001.tif]

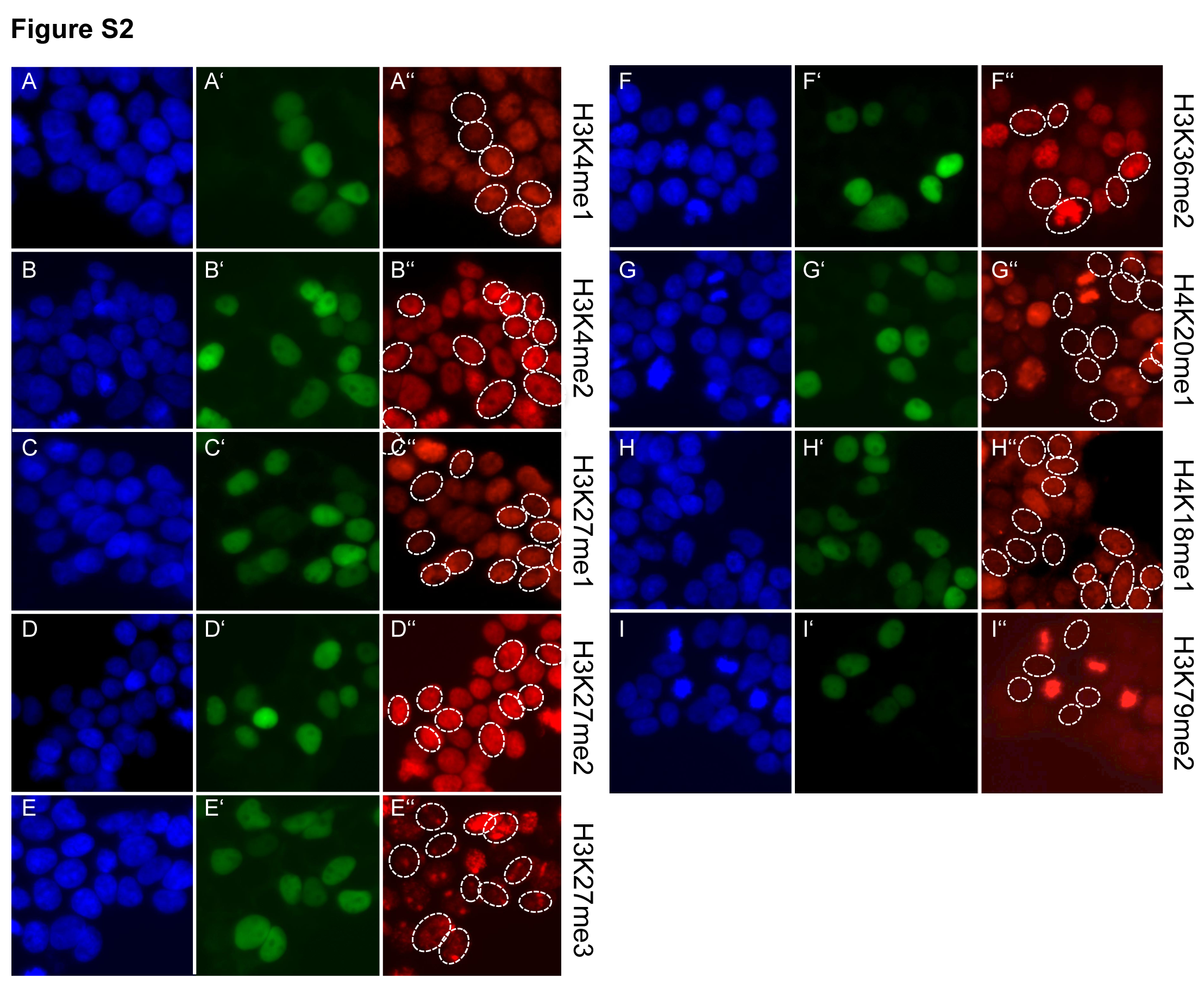

Supplement: Figure S2 — Analysis of additional methyl marks upon overexpression of JMJD1C. (TIF) [file pone.0060549.s002.tif]

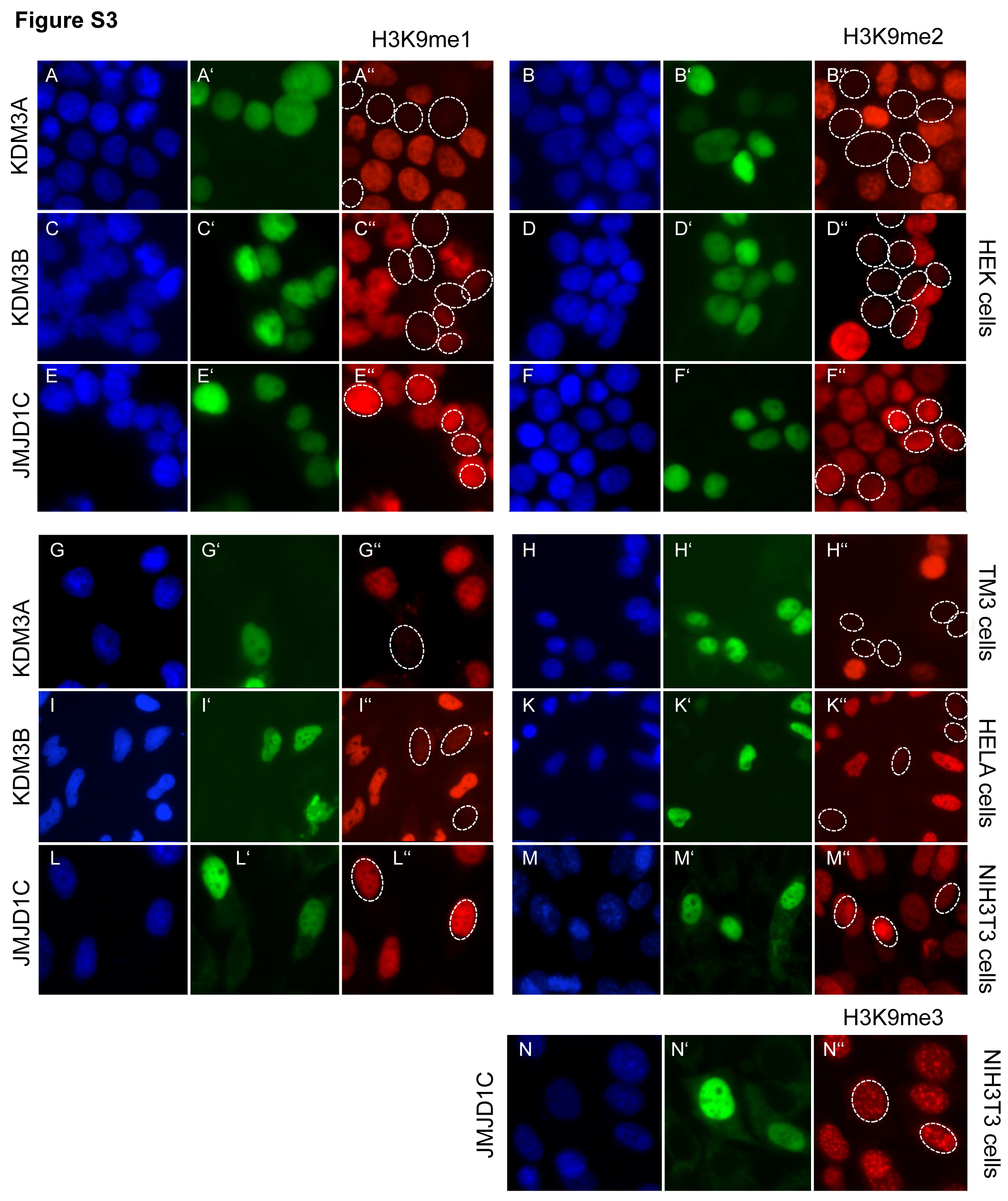

Supplement: Figure S3 — Enzymatic activity of full-length KDM3 subfamily members towards H3K9 methylation in HEK293T, HeLa, TM3 and NIH3T3 cell lines. (TIF) [file pone.0060549.s003.tif]

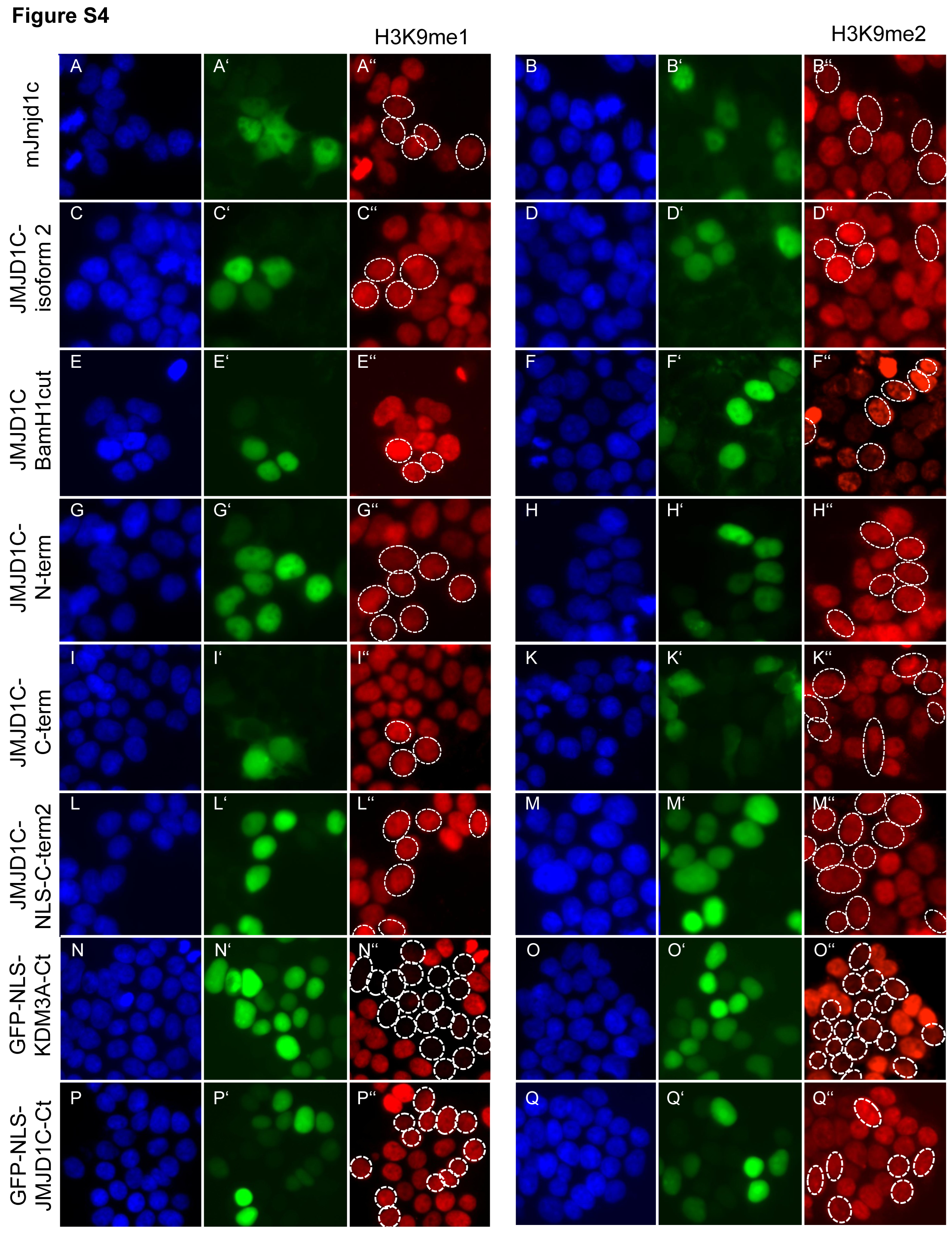

Supplement: Figure S4 — Enzymatic activity of mJmjd1c, as well as KDM3A and hJMJD1C deletion constructs towards H3K9 methylation. (TIF) [file pone.0060549.s004.tif]

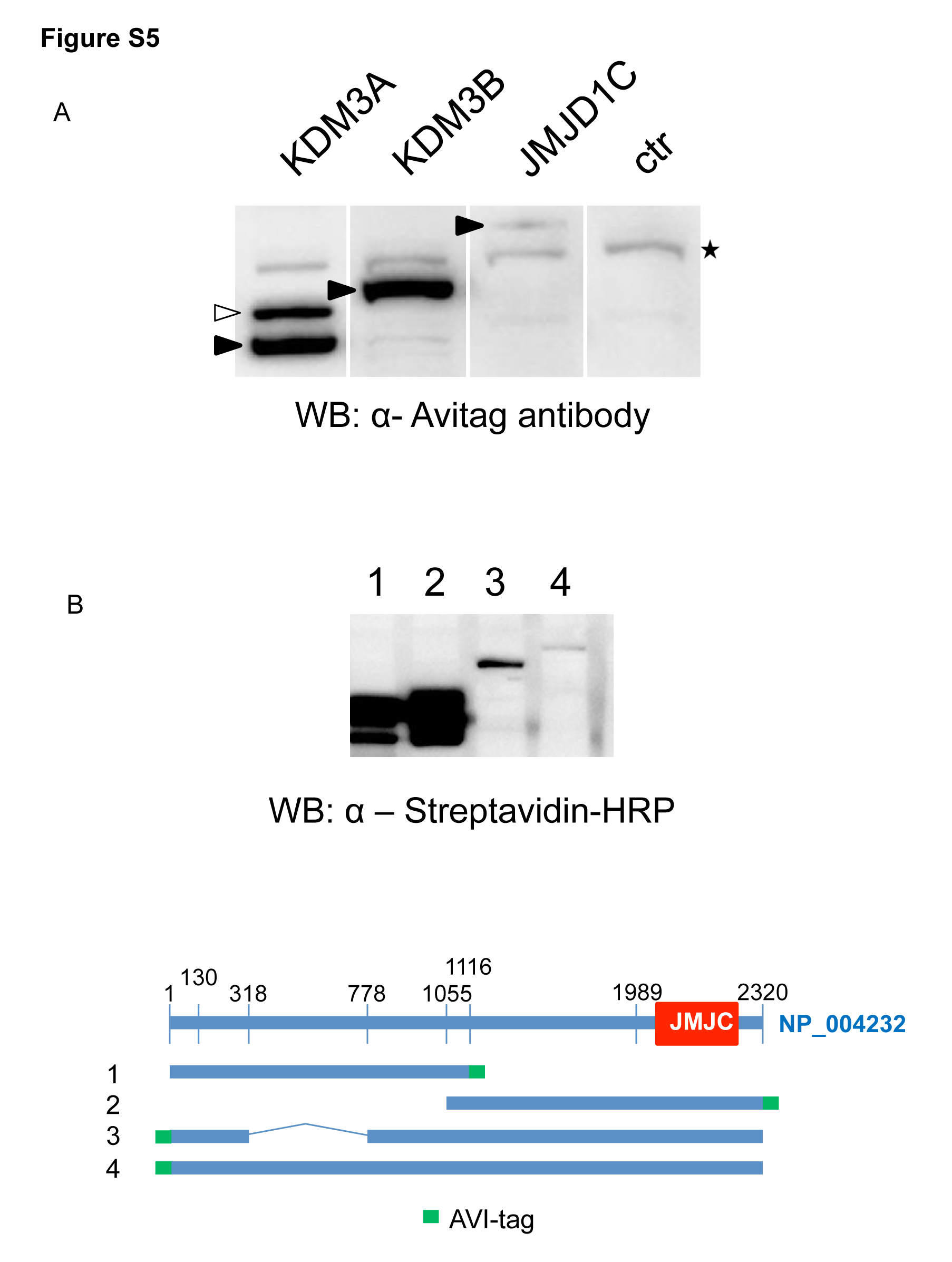

Supplement: Figure S5 — Avi-KDM3A, -KDM3B and –JMJD1C levels, including certain deletion constructs, upon overexpression in HEK293T cells. (TIF) [file pone.0060549.s005.tif]

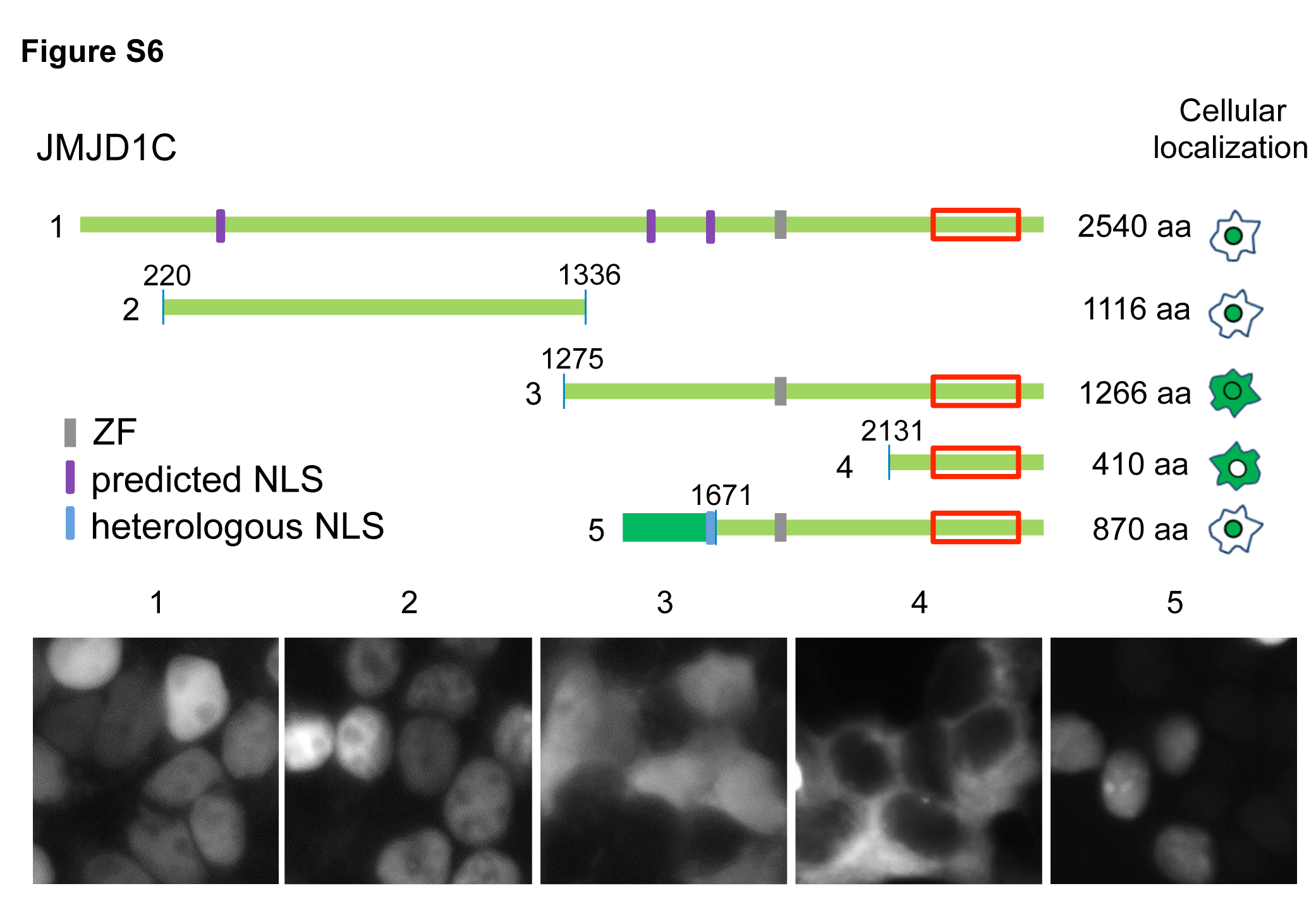

Supplement: Figure S6 — Sub-cellular localization of JMJD1C deletion constructs. (TIF) [file pone.0060549.s006.tif]

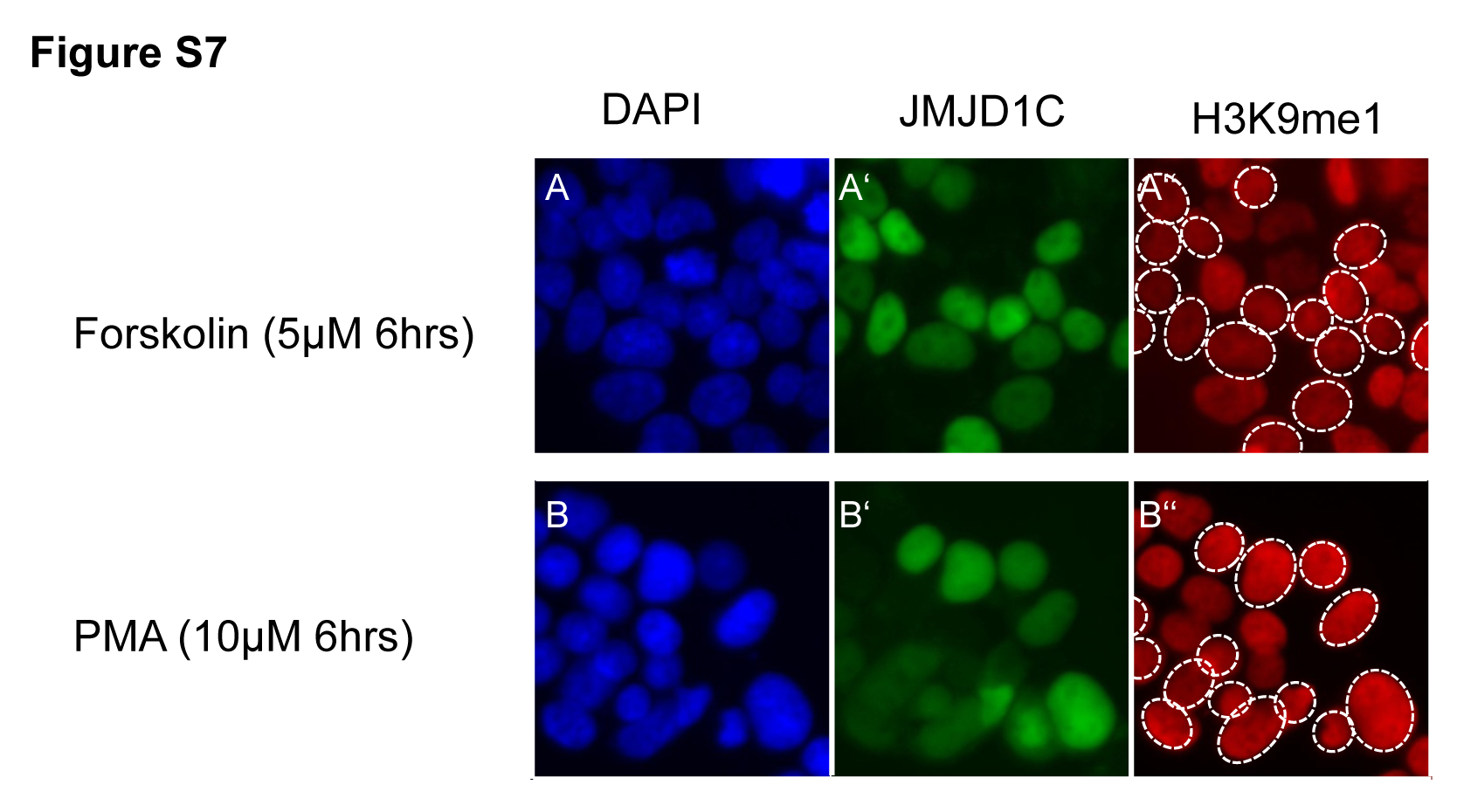

Supplement: Figure S7 — Lack of enzymatic activity of JMJD1C overexpression upon treatment with kinase activators forskolin and PMA. (TIF) [file pone.0060549.s007.tif]

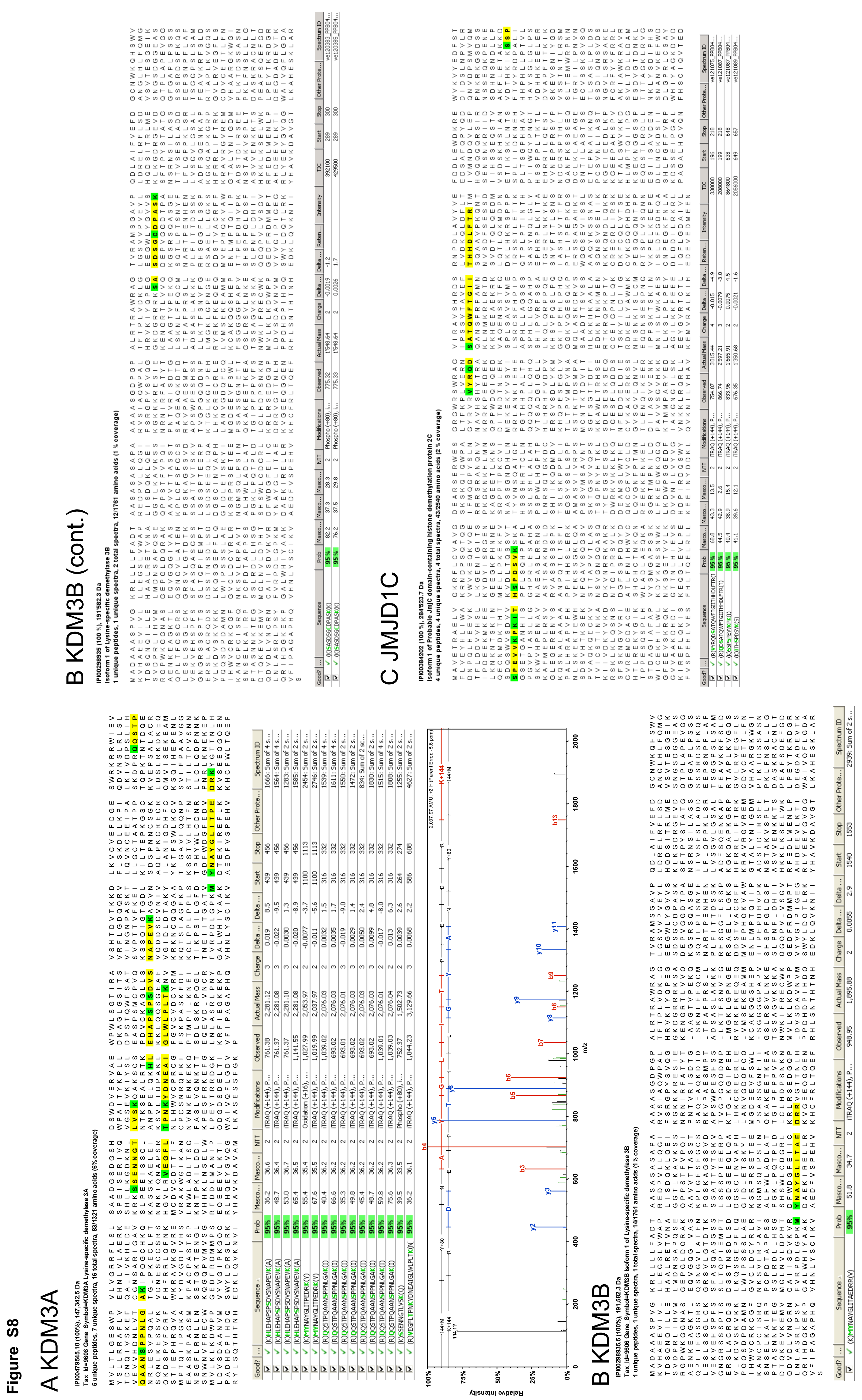

Supplement: Figure S8 — Detection of phosphorylation events in KDM3 subfamily members. (TIF) [file pone.0060549.s008.tif]

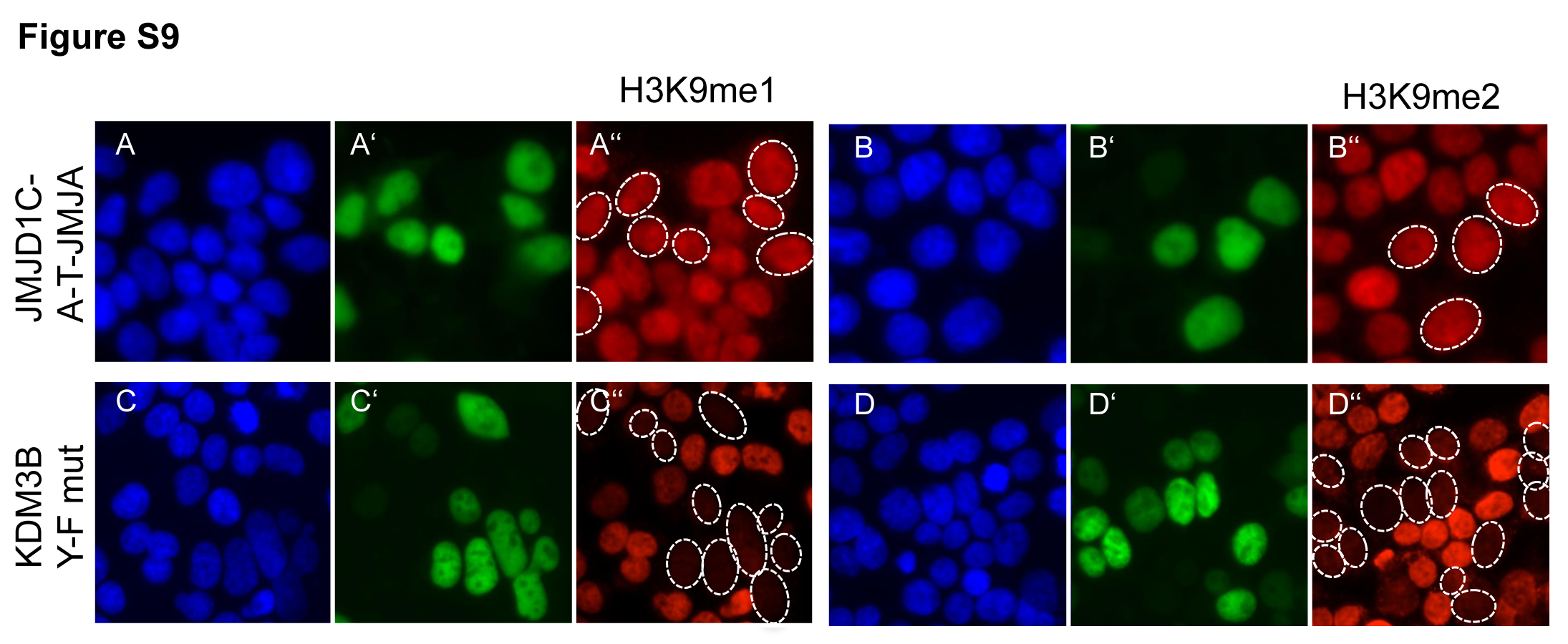

Supplement: Figure S9 — Enzymatic activity of mutated KDM3 subfamily members towards methylated H3K9. (TIF) [file pone.0060549.s009.tif]

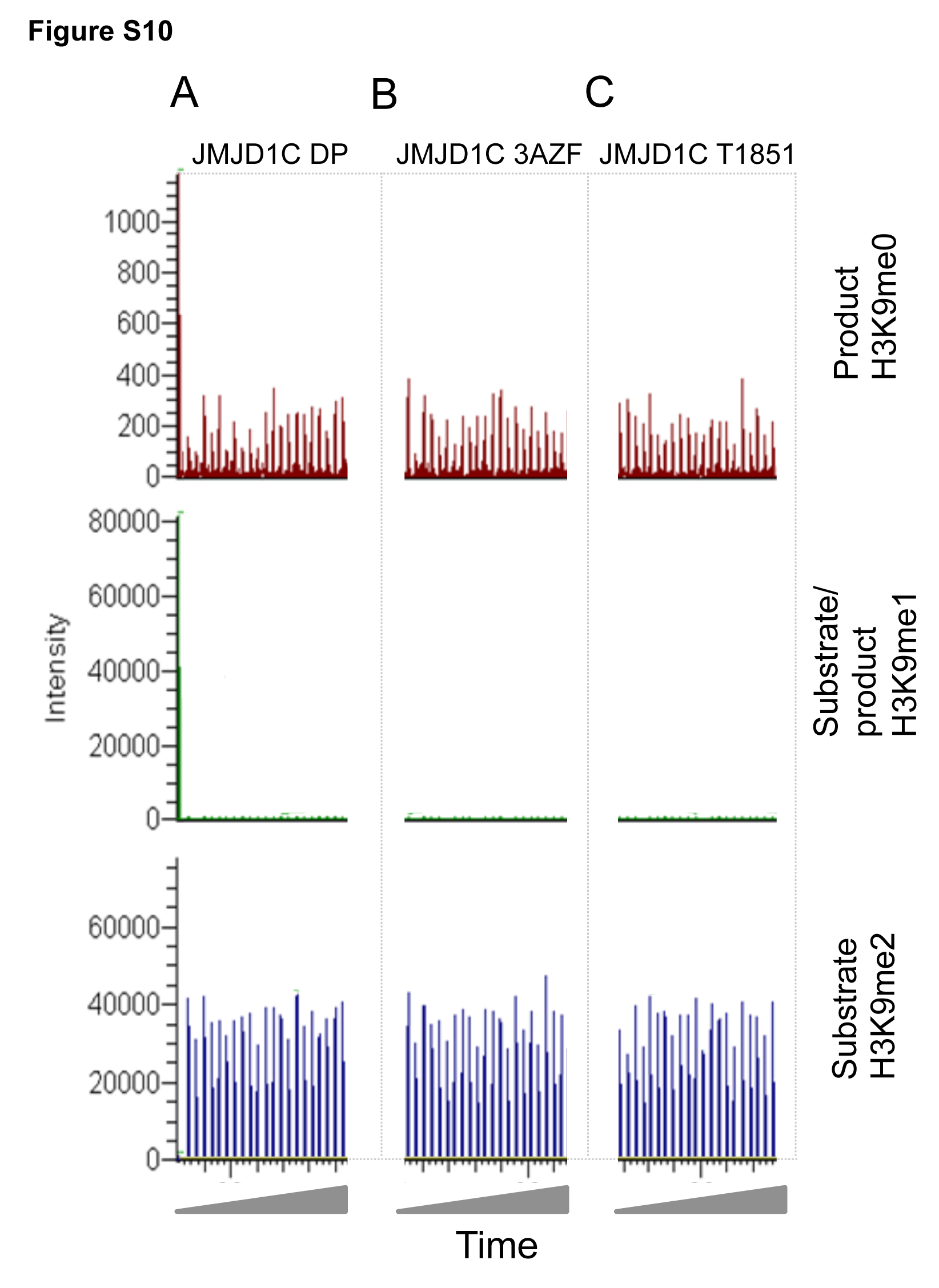

Supplement: Figure S10 — Lack of enzymatic activity of additional JMJD1C constructs in the biochemical assay. (TIF) [file pone.0060549.s010.tif]

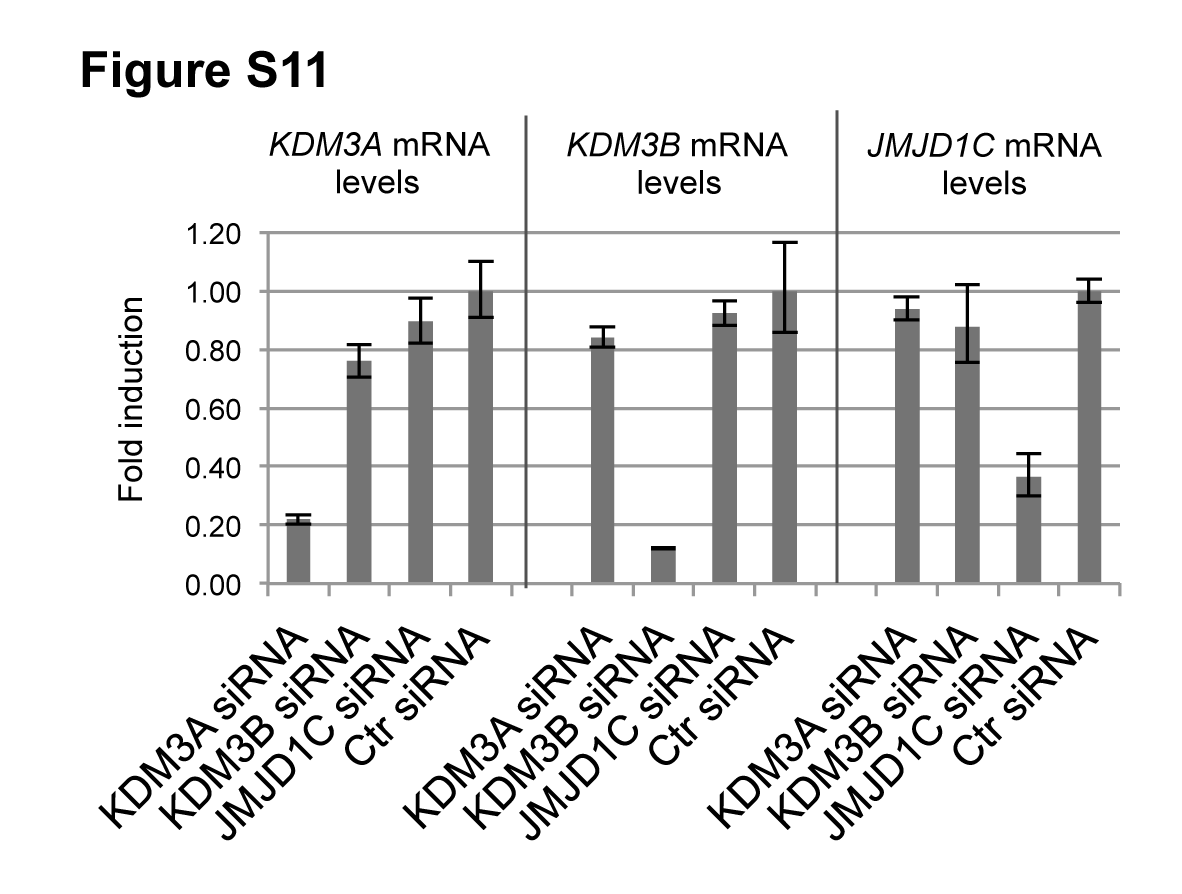

Supplement: Figure S11 — No effect on KDM3 subfamily member gene expression upon reciprocal subfamily member gene knockdown. (TIF) [file pone.0060549.s011.tif]
